# IRGM Is a Common Target of RNA Viruses that Subvert the Autophagy Network

**DOI:** 10.1371/journal.ppat.1002422

**Published:** 2011-12-08

**Authors:** Isabel Pombo Grégoire, Clémence Richetta, Laurène Meyniel-Schicklin, Sophie Borel, Fabrine Pradezynski, Olivier Diaz, Alexandre Deloire, Olga Azocar, Joël Baguet, Marc Le Breton, Philippe E. Mangeot, Vincent Navratil, Pierre-Emmanuel Joubert, Monique Flacher, Pierre-Olivier Vidalain, Patrice André, Vincent Lotteau, Martine Biard-Piechaczyk, Chantal Rabourdin-Combe, Mathias Faure

**Affiliations:** 1 Université de Lyon, Lyon, France; 2 INSERM, U851, Lyon, France; 3 Université Lyon1, UMS3444/US8, Lyon, France; 4 Université de Montpellier, CPBS, UM1, UM2, CNRS, Montpellier, France; 5 CNRS URA 3015, Institut Pasteur, Paris, France; 6 Hospices Civils de Lyon, Lyon, France; University of New Mexico, United States of America

## Abstract

Autophagy is a conserved degradative pathway used as a host defense mechanism against intracellular pathogens. However, several viruses can evade or subvert autophagy to insure their own replication. Nevertheless, the molecular details of viral interaction with autophagy remain largely unknown. We have determined the ability of 83 proteins of several families of RNA viruses (*Paramyxoviridae, Flaviviridae, Orthomyxoviridae, Retroviridae* and *Togaviridae*), to interact with 44 human autophagy-associated proteins using yeast two-hybrid and bioinformatic analysis. We found that the autophagy network is highly targeted by RNA viruses. Although central to autophagy, targeted proteins have also a high number of connections with proteins of other cellular functions. Interestingly, immunity-associated GTPase family M (IRGM), the most targeted protein, was found to interact with the autophagy-associated proteins ATG5, ATG10, MAP1CL3C and SH3GLB1. Strikingly, reduction of IRGM expression using small interfering RNA impairs both Measles virus (MeV), Hepatitis C virus (HCV) and human immunodeficiency virus-1 (HIV-1)-induced autophagy and viral particle production. Moreover we found that the expression of IRGM-interacting MeV-C, HCV-NS3 or HIV-NEF proteins *per se* is sufficient to induce autophagy, through an IRGM dependent pathway. Our work reveals an unexpected role of IRGM in virus-induced autophagy and suggests that several different families of RNA viruses may use common strategies to manipulate autophagy to improve viral infectivity.

## Author Summary

Autophagy is a highly regulated cellular degradative pathway for recycling of long-lived proteins and damaged organelles. Autophagy is also used by host cells as a defense mechanism against intracellular pathogens. Autophagy can degrade pathogens or pathogen-derived molecules trapped within specialized vesicles named autophagosomes. Viruses and viral proteins are not an exception. However, since autophagy is a conserved pathway, viruses were submitted to an evolutionary pressure that led to the selection of molecular strategies which avoid or subvert this process to promote viral replication. Nevertheless the molecular details of viral interaction with autophagy remain largely unknown. We determined the ability of 83 proteins of several families of RNA viruses (including Hepatitis C virus (HCV), human immunodeficiency virus 1 (HIV-1), Measles virus (MeV) and influenza A virus) to interact with 44 human proteins known to regulate autophagy and found that autophagy is highly targeted by RNA viruses. Strikingly, immunity-associated GTPase family M (IRGM), known for its role in autophagy against bacteria, is the most targeted autophagy protein. Its absence is detrimental for HCV, HIV-1 and MeV production. Therefore, our data show that different RNA viruses families use similar strategies to fine tune autophagy to their own benefit.

## Introduction

Macroautophagy (thereafter referred to as autophagy) is a highly regulated self-degradative mechanism for intracellular clearance and recycling of cytoplasmic contents [1]. During this process large portions of the cytoplasm are engulfed into autophagosomes that subsequently fuse with lysosomes to form acidic autolysosomes where degradation occurs. The autophagy process results from a cascade of reactions orchestrated by autophagy-related genes (*atg*) encoding ATG proteins mostly defined in yeast and for which numerous mammalian orthologs have been identified [2]. However, the function of most of these *atg* remains poorly characterized and several non *atg* mammalian genes were also described to regulate autophagy. During autophagy, the formation of an isolation membrane is initiated by class III phophatidylinositol 3-kinase (PIK3C3)/Beclin1 containing complexes [3]–[5]. The elongation of the isolation membrane involves two ubiquitin-like conjugation systems [6], [7]. In one of them, ATG12 associates with ATG5 for the formation of ATG12-ATG5-ATG16L1 molecular complexes that bind the outer membrane of the isolation membrane. In the second, LC3 is coupled with phosphatidylethanolamine to generate a lipidated LC3-II form that is integrated in both the outer and inner membranes of the autophagosome.

Whereas required at a basal level for cellular homeostasis maintenance, autophagy is used as a universal innate cell defense mechanism to fight intracellular pathogens allowing their delivery to degradative lysosomes [8], [9]. Studies involving overexpression or knock-down of *atg* have demonstrated an important role for autophagy in both innate antibacterial [10]–[12] and antiviral defense [13], [14]. Autophagy contributes to immune surveillance via cytoplasmic sampling and delivery of intracellular pathogens or components of these pathogens to endosomes and major histocompatibility complex (MHC)-II molecules rich compartments, thus promoting innate recognition by endosomal Toll-like receptors (TLR) [15] and pathogen-adaptive immune response [16]–[18], respectively. However, since autophagy is a conserved pathway, intracellular pathogens were submitted to an evolutionary pressure that led to the selection of pathogens with different molecular strategies to avoid or subvert this process to their own benefit [8].

RNA viruses include several viral species that are of major concerns in public health such as Hepatitis C virus (HCV), human immunodeficiency virus 1 (HIV-1), influenza A, Measles virus (MeV) or Dengue virus. These viruses dispose of a limited number of viral proteins to control major cellular pathways such as protein production or degradation, cell survival and evasion from host cell defense. Several RNA viruses have been shown to subvert autophagy, nevertheless few viral molecular adaptations to host autophagy have been identified [19]–[25]. HIV-1 and influenza A are two viruses that block autophagosome maturation. It has been shown that both HIV-1-NEF and influenza A-M2 proteins target Beclin1 to prevent autolysosome formation [21], [24]. The identification of new viral factors able to physically interact with autophagy-associated proteins and the characterization of their functional consequences on autophagy might provide insights on the strategies used by RNA viruses to manipulate and/or subvert this pathway.

The growing knowledge of the molecular partners underlying the execution and the regulation of the autophagy process prompted us to analyze whether this machinery is particularly targeted by RNA viruses. Using a yeast two-hybrid approach and bioinformatics analysis, we have determined how proteins from 5 different RNA virus families (*Paramyxoviridae, Flaviviridae, Orthomyxoviridae, Togaviridae and Retroviridae*) physically interact with host autophagy-associated proteins. We have found that autophagy is a functional network highly targeted by RNA virus proteins. In particular, we observed that IRGM is able to interact with proteins from 5 different RNA virus families. Although IRGM was previously reported to play an autophagy-dependent anti-bacterial function [26], [27], the mechanisms underlying the role of IRGM in autophagy remain poorly understood [27]–[29]. We found that this protein interacts with several key proteins of the autophagy process. Furthermore, we describe a role of IRGM in both virus-dependent autophagy induction and virus production. Our results suggest that different RNA virus families have a conserved way to overcome the host autophagy pathway in order to lead to a successful infection.

## Results

### RNA viruses interact with multiple autophagy-associated proteins

**Figure 1:**
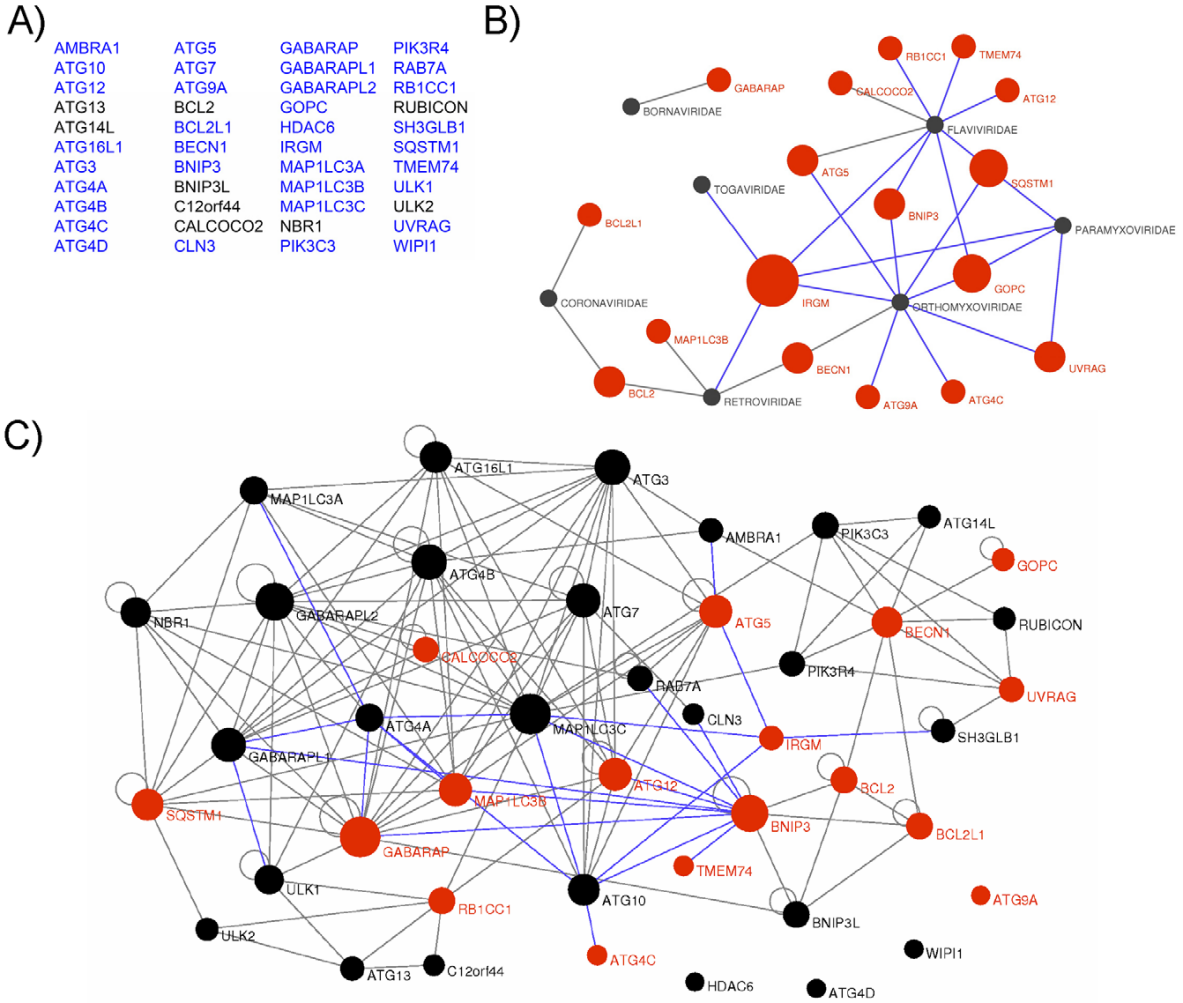
Targeting of the autophagy network by RNA viruses. (A) List of the 44 autophagy-associated proteins considered in this study. Proteins represented in blue were used in yeast two-hybrid arrays. (B) Autophagy-associated proteins are targeted by RNA viruses. Graphical representation of ppi network of autophagy proteins and RNA virus proteins. Red nodes represent autophagy-associated proteins. Dark grey nodes represent RNA viral families. Blue edges represent novel ppi and gray edges represent ppi retrieved from the literature. The width of the red nodes is proportional to the number of viral families targeting the autophagy-associated proteins. (C) RNA viruses targeted human autophagy network. Graphical representation of the targeted human autophagy network. Each node represents one autophagy-associated protein. Black nodes are autophagy-associated proteins with unknown RNA virus interactions and red nodes represent autophagy-associated proteins targeted by at least one RNA virus protein. Blue edges represent novel ppi and gray edges represent ppi retrieved from the literature. The width of the nodes is proportional to the number of autophagy-associated proteins directly interacting with the considered protein.

To gain an insight on the molecular mechanisms by which RNA viruses modulate the autophagy process, we tested whether viral proteins belonging to various strains of 5 different viral families (*Paramyxoviridae,* MeV, Mumps virus, Nipah virus; *Flaviviridae*, HCV, Dengue virus, West Nile virus, Tick borne encephalitis virus and Kyasanur forest disease virus; *Orthomyxoviridae*, Influenza A virus; *Retroviridae,* HIV-1; *Togaviridae*, Chikungunya virus) were able to physically interact with human autophagy proteins. We established a list of 44 autophagy-associated proteins closely involved in human autophagy, based on data available from the literature showing the functional involvement of the proteins in autophagy using either short interfering (si)-RNA shutting down the expression of the considered protein or protein overexpression (Figure 1A and Table S1). Proteins that despite their crucial involvement in autophagy are broad signalling regulators of different cellular pathways were not included in our study. From this list 35 autophagy-associated proteins (Figure 1A in blue and Table S2) were available to be tested pairwise in a yeast two-hybrid array against 80 different viral proteins (Table S3). We found 42 new protein-protein interactions (ppi) between viral proteins and autophagy-associated proteins to which were added the 10 ppi between RNA virus proteins and autophagy-associated proteins described in the literature (Figure 1B and Table S4). We further tested the ability of IRGM, GOPC and SQSTM1, the most common RNA virus targets, to interact with three different HIV-1 proteins (Table S3/S4). Overall, among the 17 different autophagy-associated proteins we found to be targeted by RNA virus proteins, 9 proteins interact with one RNA virus family, 5 interact with 2 different families (BECN1, BCL2, UVRAG, ATG5, BNIP3), 2 interact with 3 different RNA virus families (SQSTM1 and GOPC) and one autophagy-associated protein, IRGM, is a common target of 5 different families of RNA viruses (*Paramyxoviridae, Flaviviridae, Orthomyxoviridae*, *Togaviridae, Retroviridae*) (Figure 1B, Figure S3 and Table S4).

### RNA viruses target human autophagy-associated proteins that form a highly interconnected protein network

Here, our results show that RNA virus proteins interact with more than 35% of the autophagy-associated proteins suggesting that autophagy is a widely targeted functional network. This targeting is highly significant as compared to the targeted human proteome counterpart (exact Fisher test, p value <2,2×10^−16^). Nevertheless, a defined protein may play important roles in several cellular functions; therefore a viral interaction with a particular autophagy protein does not preclude an effect in a precise pathway. To understand whether the proteins targeted by RNA viruses are particularly dedicated to the autophagy process or may be important to connect this conserved cellular function to other cellular processes, we have established a comprehensive map of ppi between the 44 autophagy-associated proteins (Figure 1C, each node represents one autophagy-associated protein). We first determined this network by systematically testing pairwise 35 autophagy-associated proteins (proteins in blue in Figure 1A) in a yeast two-hybrid array and incremented our own set of data with those from the literature to build the autophagy network [30] (Figure 1C, Table S5). Overall, the human autophagy network is composed of a total of 150 ppi, among them we identified 23 novel intra-autophagy network interactions (Figure 1C, blue edges). Interestingly, the human autophagy network appears as a highly interconnected cellular network, with a single connected component of 40 proteins, and only four isolated proteins (ATG4D, ATG9A, WIPI1 and HDAC6) for which to date no protein interaction was identified within the network. This interconnectivity is significantly higher than the theoretical interconnectivity computed from resampled subnetworks (resampling test, n = 10000, p value <10^−4^, Figure S1). This high significance supports the functional consistency of the initially chosen group of 44 autophagy-associated proteins.

### An important fraction of autophagy-associated proteins are both central to the autophagy network and highly connected to other cellular pathways

**Figure 2:**
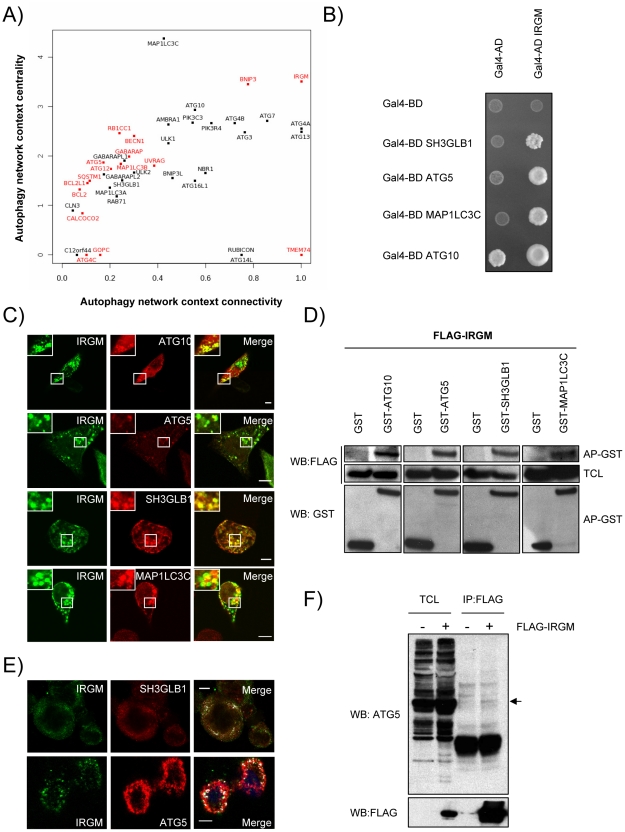
IRGM, a protein that is highly targeted by RNA viruses, co-localizes and interacts with several autophagy-associated proteins. (A) Autophagy-associated proteins contribute differently to the autophagy network. Autophagy-associated proteins are plotted according to their context connectivity and context centrality. Autophagy context connectivity (x axis) is the ratio of interaction in autophagy network over the interaction in the whole human protein interaction network. Autophagy context centrality (y axis) is the ratio of betweenness (log normalized values) in autophagy network over the betweenness in the human protein interaction network. A protein with high degree and high betweenness ratios is respectively defined as highly devoted and highly central in the autophagy context. Proteins in red represent autophagy-associated proteins targeted by at least one RNA virus protein. The four proteins that are not connected to the autophagy network are not represented. (B) IRGM interacts with ATG10, ATG5, SH3GLB1 and MAP1LC3C. IRGM was tested by yeast two-hybrid against 35 different autophagy-associated proteins. Positive interactions with ATG10, ATG5, SH3GLB1 and MAP1LC3C were found. A reduced yeast two-hybrid matrix containing positive interactions and the appropriate empty vector controls is shown. One experiment representative of three is shown. (C) IRGM co-localizes with autophagy-associated proteins. GFP-IRGM was expressed in HeLa cells together with FLAG-ATG10, FLAG-ATG5, FLAG-SH3GLB1 or FLAG-MAP1LC3C. Fixed cells were then stained with an anti-FLAG antibody and GFP-IRGM and FLAG-tagged proteins co-localisation was visualized on merged images by confocal microscopy. Scale bars, 5 µM. (D) IRGM interacts with autophagy-associated proteins. HEK293T cells were co-transfected with GST and GST-tagged expression vectors encoding the indicated proteins and FLAG-IRGM. Interaction was assayed by co-affinity purification (AP) using glutathione-sepharose beads. FLAG-IRGM was detected using anti-FLAG antibody after (AP-GST, WB: FLAG) and before (total cell lysate-TCL, WB: FLAG) co-AP. GST alone and GST-tagged proteins were detected by using anti-GST antibody (AP-GST, WB: GST). One experiment representative of two is shown. (E) Endogenous IRGM co-localizes with endogenous SH3GLB1 and ATG5 in MeV infected cells. HeLa cells were infected with MeV Edmonston (MOI = 1) for 24 hrs. Cells were fixed in acetone and both IRGM and/or SH3GLB1 or ATG5 were detected using specific antibodies. IRGM/SH3GLB1 or IRGM/ATG5 protein co-localisation was visualized on merged images obtained by confocal microscopy. Scale bars, 5 µM. (F) Endogenous ATG5 interacts with FLAG-IRGM. HeLa cells were transfected or not with FLAG-IRGM encoding vector and infected with MeV (MOI = 1) 24 hrs post-transfection. Cells were lysed 24 hrs post-infection. Flag-tagged IRGM was immunoprecipitated and endogenous ATG5 binding was detected by western blot (top panel). Overexpression and immunoprecipitation of FLAG tagged IRGM was confirmed by a western blot using (bottom panel).

The 44 proteins of the autophagy network do not function in isolation but interact with roughly at least 450 other cellular proteins within the whole human protein interaction network [30]. To analyze the relative functional contribution of each of the 44 autophagy-associated proteins to the autophagic process without *a priori,* we determined their respective connectivity and centrality within the autophagy network and within the whole human protein interaction network (Figure 2A and Table S6) [31]. The connectivity or degree of a protein is the number of direct interacting partners of this protein We have defined the autophagy context-dependent connectivity as the ratio of the protein connectivity within the autophagy network over those within the whole human protein interaction network [31] (Figure 2A and Figure S2A). This highlights 10 different proteins that have more than two thirds of their interactions inside the autophagy network, suggesting that they might be particularly dedicated to the autophagy network (Figure 2A, x axis>0.66). Examples include several different ATGs (ATG3, ATG4A/B, ATG7) but also BNIP3 and IRGM. Interestingly, half of the autophagy-associated proteins have less than one third of their interactions within the autophagy network i.e. are essentially connected outside this network (Figure 2A, x axis<0.33). Examples include BCL2, ATG5, ATG12 or BECN1, indicating that an important fraction of autophagy-associated proteins might be involved in the crosstalk between autophagy and other cellular pathways.

Although the number of connections of a protein is relevant, the flux of information passing through this protein, illustrated by its centrality or betweenness, is another critical parameter that determines the influence of a protein in a network. We have defined the autophagy context dependent-centrality as the ratio of the protein betweenness within the autophagy network over those within the whole human protein interaction network [31] (Figure 2A and Figure S2B). Interestingly, most of the 44 autophagy-associated proteins appear as essential components of the autophagy system (Figure 2A, y axis>1). Among them were found most of the ATGs, ULK1, BECN1, AMBRA1, PIK3C3 and PIK3R4, BNIP3 and IRGM that exhibit increased betweenness values in the autophagy network compared to the whole human interactome.

### IRGM is highly targeted by RNA viruses

Our results show that RNA viruses mainly target proteins that although central to this functional network connect autophagy to other cellular functions (Figure 2A, y axis>1, targeted proteins represented in red). Indeed, with the exception of three proteins (IRGM, BNIP3 and TMEM74), all RNA virus-targeted autophagy-associated proteins have more than 60% of their interactions that take place out of the autophagy network (Figure 2A, x axis<0.4 targeted proteins represented in red). Strikingly, IRGM is noteworthy by being commonly targeted by 5 different families of RNA viruses (Figure 1B and Figure S4) and by making all its cellular protein interactions within the autophagy network (Figure 2). These results prompted us to investigate whether IRGM and the autophagy interactors we identified by yeast two-hybrid (Figure 2B) could associate in mammalian cells. In co-transfected human HeLa cells, we first found that IRGM co-localizes with ATG10, ATG5, SH3GLB1 and MAP1LC3C (Figure 2C). Furthermore, physical interactions between IRGM and each of these autophagy-associated proteins were confirmed by GST-co-affinity experiments in human cells (Figure 2D). We found that endogenous IRGM colocalizes with endogenous SH3GLB1 and ATG5 (Figure 2E), and that a small fraction of endogenous ATG5 interacts with overexpressed IRGM but not with SQSTM1 (Figure 2F and Figure S5). Together, our results show that IRGM can interact with several autophagy-associated proteins.

### Autophagosome formation during MeV, HCV and HIV-1 infections depends on IRGM

IRGM being both particularly targeted by RNA viruses and interacting with several autophagy-associated proteins, prompted us to test whether this protein was involved in virus-induced autophagy. We have found that IRGM interacts with MeV (*Paramyxoviridae* family), HCV (*Flaviviridae* family), influenza A (*Orthomyxoviridae* family) and HIV-1 (*Retroviridae* family) proteins (Figure 1B, Figure S4 and Table S4), four viruses described to induce autophagosome accumulation upon infection [19], [21], [24], [32]. To determine whether IRGM is involved in autophagosome formation observed upon MeV, HCV and influenza A infections we have abrogated IRGM expression using specific si-RNA (Figure S6A–G), prior to infection in GFP-LC3-HeLa cells, GFP-LC3-Huh 7.5 or GFP-LC3-A549 cells, respectively. IRGM mRNA (Figure S6F) and endogenous protein (Figure S6C–E, G) was detected in cell lines used in our studies. The level of expression of the endogenous protein is specifically decreased in cells treated with si-IRGM (Figure S6D/E), as previously observed [28].

**Figure 3:**
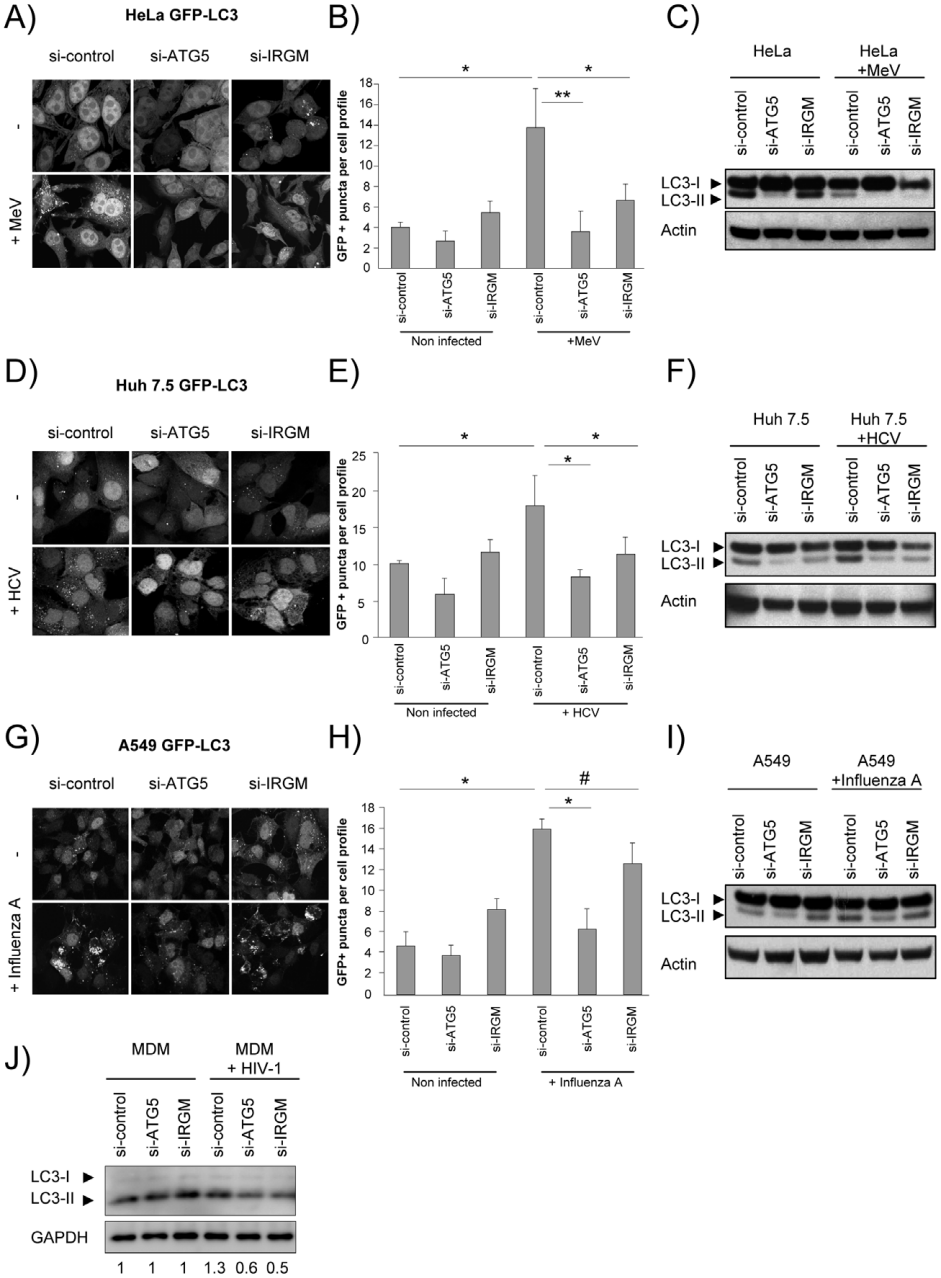
MeV, HCV and HIV-1-induced autophagosome accumulation is dependent on IRGM. (A–C) MeV-induced autophagy is dependent on IRGM. GFP-LC3-HeLa (A–B) or HeLa cells (C) were treated with si-control, si-ATG5 or si-IRGM and either left uninfected or infected with MeV Edmonston (MOI = 3) for 24 hrs. Autophagy was monitored either by evaluating the number of GFP-LC3+ vesicles per cell profile by confocal microscopy (A, B) or by detection of LC3-I and LC3-II by western-blot (C). Representative profiles for each condition (A) and the corresponding graph representing the number of GFP-LC3+ vesicles per cell profile (B) are shown, error bars, mean ± SD of three independent experiments. (C) One experiment representative of three is shown. (D–F) HCV-induced autophagy is dependent on IRGM. GFP-LC3-Huh7.5 (D–E) or Huh7.5 (F) cells were treated with the indicated si-RNA and either left uninfected or infected with HCV JFH-1 (MOI = 1) for 24 hrs. Autophagy was monitored as above. (E) Error bars, mean ± SD of three independent experiments. (F) One experiment representative of two is shown. (G–I) Influenza A-induced autophagy is not impaired by IRGM absence. GFP-LC3-A549 (G–H) or A549 (I) cells were treated with the indicated si-RNA and either left uninfected or infected with influenza A/H1N1/New Caledonia (MOI = 1) for 24 hrs. Autophagy was monitored as above. (H) Error bars, mean ± SD of two independent experiments. (I) One experiment representative of two is shown. (J) HIV-1-induced autophagy is dependent on IRGM. Monocyte-derived macrophages (MDM) were treated with the indicated si-RNA and were either left uninfected or infected with HIV-1 for 24 hrs. LC3-I and LC3-II detection was carried out by western-blot as in C, F, I. One experiment representative of two is shown with the quantification number representing the intensity of LC3-II/GAPDH bands normalized to the uninfected condition. Student's t test; * p<0.05; ** p<0.01; # p>0.05.

Autophagosomes were monitored by tracking the formation of GFP-LC3-labelled structures representing LC3-II-containing autophagosomes. The reduced expression of ATG5 induced by si-RNA was used as a control for autophagy extinction (Figure S7). First, we found that the reduced expression of IRGM did not significantly modulate ongoing autophagy in each tested cell line (Figure 3A/B, D/E, G/H). Second, as previously reported, we found upon MeV (Figure 3A/B), HCV (Figure 3D/E) and influenza A (Figure 3G/H) infections an increased number of autophagosomes. Interestingly, the inhibition of expression of either IRGM or ATG5 prevented the increase of autophagosomes induced by MeV and HCV (Figure 3A/B/D/E). However, contrarily to the reduced expression of ATG5, the inhibition of expression of IRGM did not prevent significantly autophagosome accumulation upon influenza A infection (Figure 3G/H).

We further tested the requirement of IRGM on MeV, HCV, influenza A and HIV-1-dependent autophagy modulation by monitoring the conversion of LC3-I (cytosolic form) to LC3–II (autophagosome-bound lipidated form) by western blot in HeLa, Huh 7.5, A549 cells and human monocyte-derived macrophages (MDM), respectively. Without infection, we found that si-IRGM-treated cells do not display modulation of the total amount of LC3-II when compared to si-control treated cells (Figure 3C/F/I/J). We next found that MeV infection did not lead to an increased amount of LC3-II in si-control treated cells but instead a decrease, when compared with uninfected si-control-treated cells. This results might be the consequence of the increase of the autophagy flux induced upon MeV infection leading to the formation of productive autolysosomes, as we already reported [32]. Nevertheless, diminished IRGM expression reduced MeV-induced LC3-II amount, similarly to si-ATG5 treatment (Figure 3C). HCV, influenza A and HIV-1 were all reported to inhibit autophagosome maturation [21], [24], [33]. We found that infection with these viruses lead to an increased amount of LC3-II, when compared to uninfected cells. Moreover, the diminished expression of IRGM reduced HCV and HIV-1-increased LC3-II amount, similarly to si-ATG5 treatment (Figure 3F/J), whereas it has no effect on influenza A infection (Figure 3I).

Finally, we determined autophagy modulation during MeV, HCV, influenza A and HIV-1 infections in the presence of bafilomycin A1 (BAF), which inhibits acidification of the autolysosomes (Figure S8). We found that upon BAF treatment the total number of GFP-LC3 dots in MeV-infected cells was further increased when compared with untreated MeV-infected cells, and no further increased was observed upon HCV or influenza A infections (Figure S8A–F). Upon HIV-1 infection a slight increase of the total amount of LC3-II was detected in BAF-treated cells when compared with uninfected cells (Figure S8G). Interestingly, the reduced expression of IRGM prevented the accumulation of autophagosomes upon MeV, HCV or HIV-1 infections, but not upon influenza A virus infection in BAF-treated cells (Figure S8).

Thus, altogether these results indicate that IRGM is involved in MeV, HCV and HIV-1-mediated autophagosome accumulation.

### IRGM modulates MeV, HCV and HIV-1 infectious particle formation

**Figure 4:**
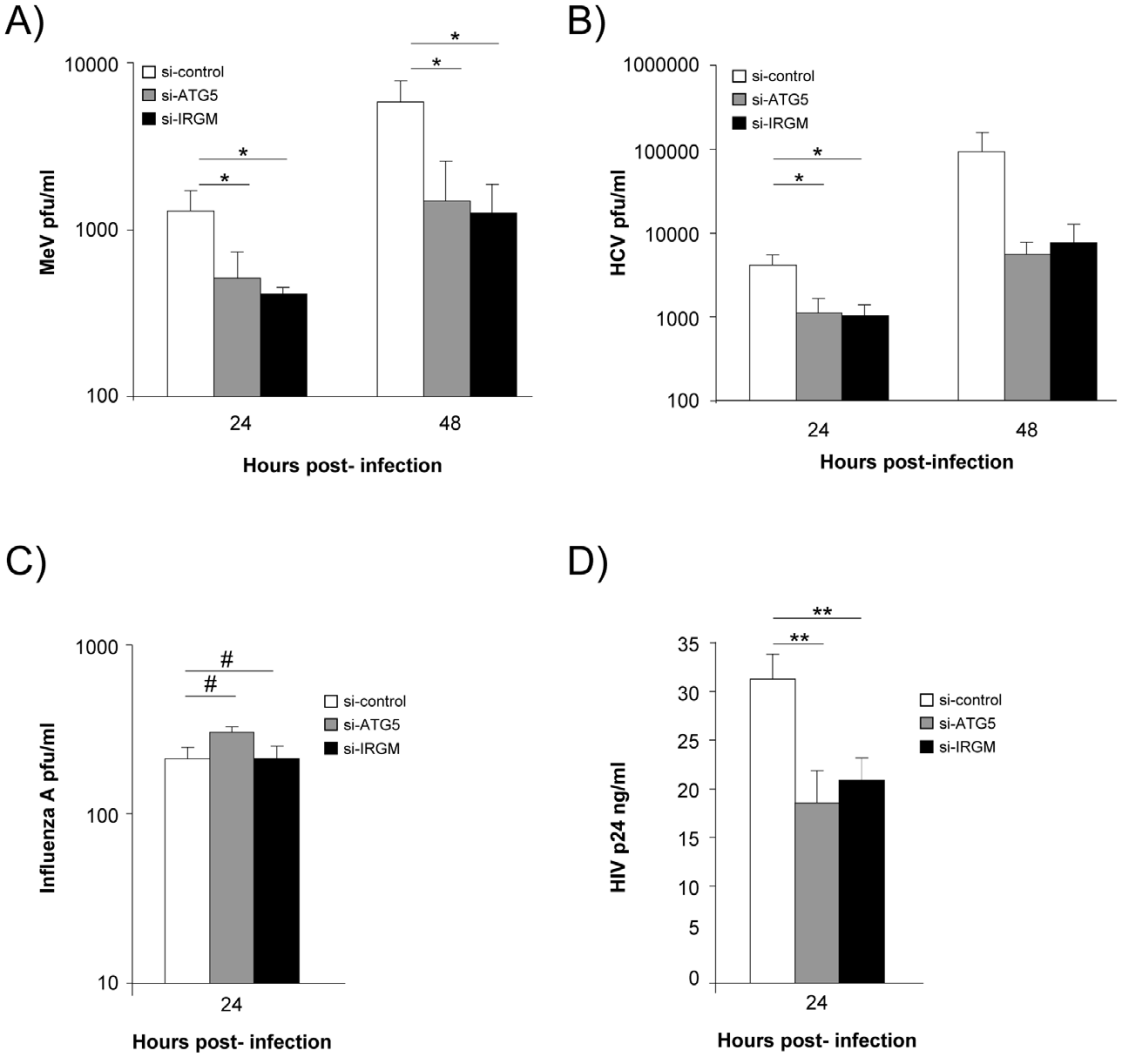
IRGM reduced expression efficiently decreases MeV, HCV and HIV-1 viral particles production. (A) IRGM is necessary for MeV infectious particle production. HeLa cells were treated with the indicated si-RNAs. Cells were infected with MeV (MOI = 3) for 24 or 48 hrs. Results display average total infectious titers expressed as plate-forming units (pfu)/ml. Error bars, mean ± SD of three independent experiments. (B) IRGM is required for HCV JFH-1 infectious particle production. Huh-7.5 cells were treated with the indicated si-RNAs prior to cell infection with HCV JFH-1 (MOI = 1). The infectivity of the virus-containing cell supernatants was determined at 24 and 48 hrs post-infection. Results display average infectious titers. Error bars, mean ± SD of respectively three (24 hrs) or two (48 hrs) independent experiments. (C) IRGM-reduced expression does not modulate influenza A infectious particle production. A549 cells were treated with the indicated si-RNAs prior to cell infection with Influenza A/H1N1/New Caledonia (MOI = 0.1). The infectivity of the virus-containing cell supernatants was determined at 24 hrs post-infection. Results display average infectious titers. Error bars, mean ± SD of two independent experiments. (D) IRGM-reduced expression modulates HIV-1 production. MDM were treated with the indicated si-RNAs prior to cell infection with HIV-1. The infectivity was determined at 24 hrs post-infection. Results correspond to one experiment in triplicate, representative of four independent experiments. Student's t test; * p<0.05; ** p<0.01; # p>0.05.

We next evaluated whether IRGM could modulate MeV, HCV, influenza A and HIV-1 infectious particle formation. To this end, we have impaired IRGM expression by si-RNA, infected HeLa cells, Huh 7.5 cells, A549 cells or human MDM with the appropriate viruses and evaluated its effect on viral particle formation. Looking at MeV infectivity, we first found that shutting down the expression of the autophagy essential gene ATG5 impaired the production of infectious particles by more than 65%, indicating that MeV hijacks autophagy to its own benefit (Figure 4A). Importantly, MeV infectious particle production was equally compromised in absence of IRGM expression (Figure 4A). Similar results were obtained concerning HCV infectivity for which the involvement of autophagy in the formation of viral particles was previously reported [20]. Interestingly we found that the absence of IRGM compromised the production of infectious HCV particles by more than 70%, similarly to the absence of ATG5 (Figure 4B). In contrast, the reduced expression of IRGM does not impair influenza A particle production (Figure 4C). Down regulation of ATG5 has also no influence on the viral production, as previously reported [21]. Finally, it was recently shown that autophagy modulates HIV production in human MDM [24], [34]. We found here that absence of IRGM compromised the production of HIV-1 particles by more than 30%, similarly to the reduced expression of ATG5 (Figure 4D). Overall our results show that IRGM is involved in the production of viral particles of at least three different RNA viruses, MeV, HCV and HIV-1.

### MeV-C, HCV-NS3 and HIV-NEF proteins interact with IRGM and induce autophagosome accumulation via a molecular process involving this protein

**Figure 5:**
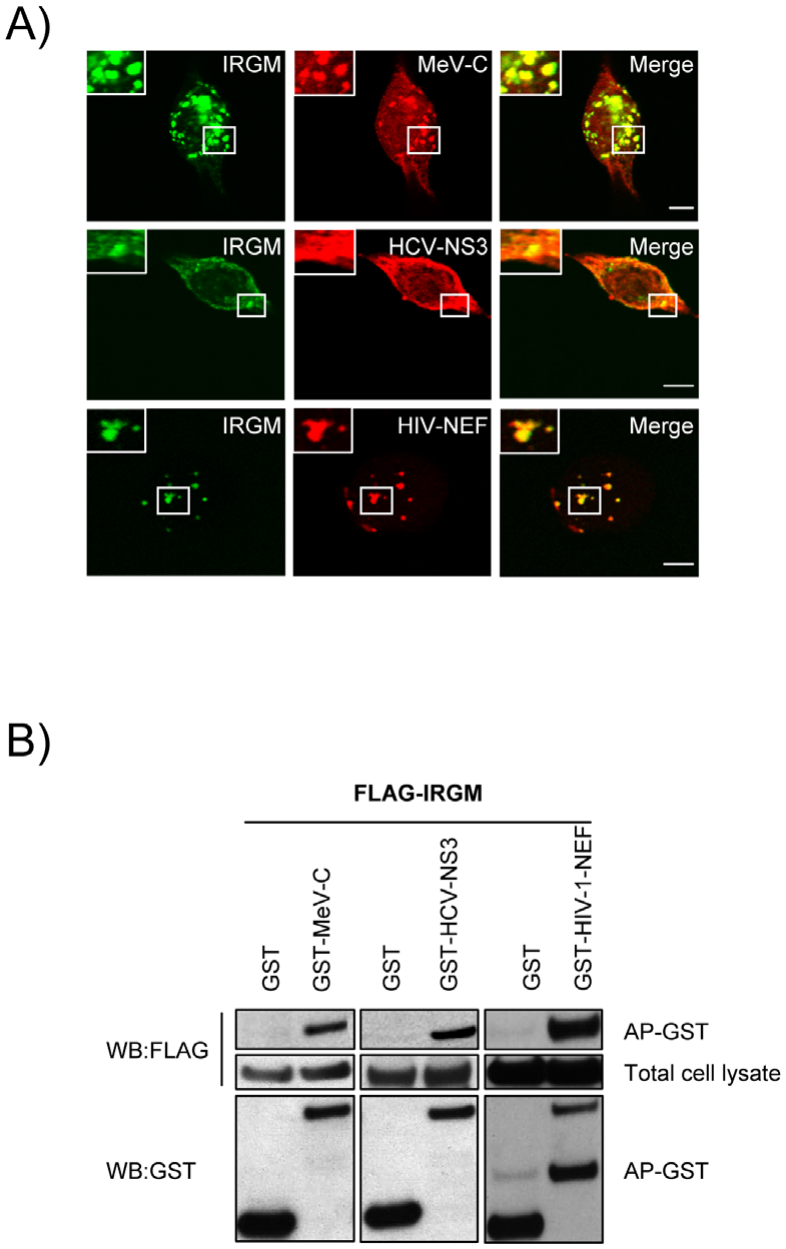
Co-localisation and physical interaction of IRGM with MeV-C, HCV-NS3 and HIV-NEF proteins. (A) IRGM co-localises with MeV-C, HCV-NS3 and HIV-NEF. GFP-IRGM was expressed in HeLa cells together with FLAG-MeV-C or FLAG-HCV-NS3 or FLAG-HIV-NEF. Co-localisation between GFP-IRGM and FLAG-tagged proteins was visualized as in figure 2B. Scale bars, 5 µM. (B) IRGM physically interacts with MeV-C, HCV-NS3 and HIV-NEF. HEK-293T cells were co-transfected with expression vectors encoding FLAG-IRGM and GST alone or GST fused to the indicated viral proteins. Co-purification of IRGM with viral proteins was assessed as in figure 2C. One experiment representative of two is shown.

To get a deeper insight on the molecular mechanisms underlining IRGM's role in both virus induced autophagy and viral particle formation we have first analysed whether IRGM and its putative viral MeV, HCV or HIV interactors could associate in human cells. In co-transfected human HeLa cells, we found that IRGM co-localizes with MeV-C, HCV-NS3 and HIV-NEF, (Figure 5A). Furthermore, we confirmed the physical interaction between IRGM and MeV-C, HCV-NS3 and HIV-NEF using GST-co-affinity experiments (Figure 5B). Thus IRGM interacts with proteins from several different RNA virus families.

**Figure 6:**
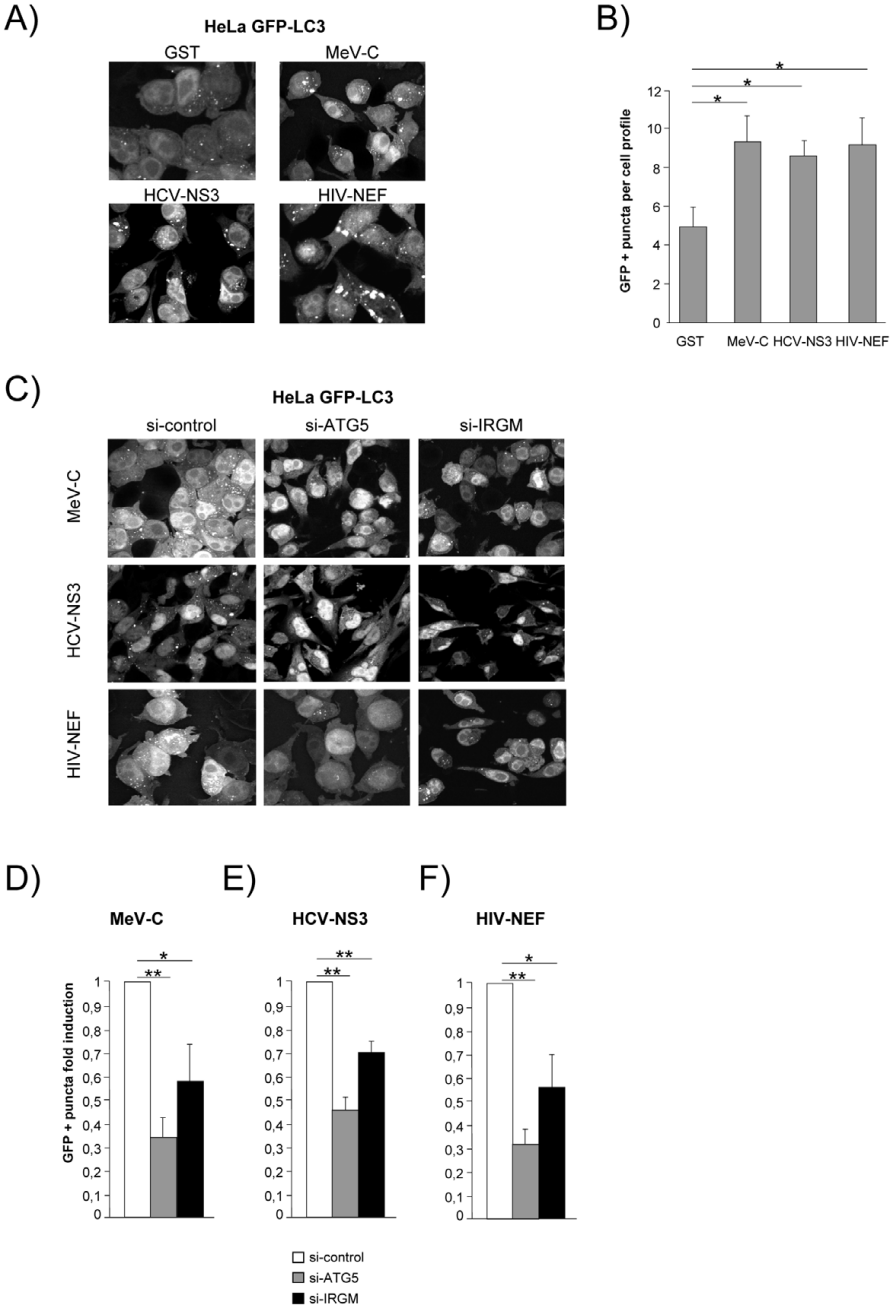
MeV-C, HCV-NS3 and HIV-NEF proteins modulate autophagy via IRGM. (A–B) Overexpression of MeV-C, HCV-NS3 and HIV-NEF modulates autophagosome formation. (A–B) GFP-LC3 HeLa cells were transfected with a GST encoding vector (control) or a vector encoding MeV-C, HCV-NS3 and HIV-NEF proteins. Twenty four hours post transfection the number of autophagic vesicles was determined by confocal microscopy. Representative profiles for each condition (A) and the corresponding graph representing the number of GFP-LC3+ vesicles per cell profile (B) are shown, error bars, mean ± SD of three independent experiments. (C–F) MeV-C, HCV-NS3 and HIV-NEF modulate autophagosome formation partly via IRGM. (C) GFP-LC3 HeLa cells were treated with si-control, si-ATG5 or si-IRGM 24 hrs prior transfection with vector encoding for MeV-C, HCV-NS3 or HIV-NEF. After an additional 24 hrs, cells were fixed and the number of autophagosome was determined by confocal microscopy. Representative profiles for each condition (C) and the corresponding graph representing the number of GFP-LC3+ vesicles per cell profile (D–F) are shown, error bars, mean ± SD of three independent experiments. Student's t test; * p<0.05; ** p<0.01.

We then determined whether the viral proteins able to interact with IRGM, could modulate autophagosome formation. To this end, MeV-C, HCV-NS3 or HIV-NEF were expressed in GFP-LC3-HeLa cells and autophagy analyzed by tracking GFP+ autophagosomes. We found that each of these viral proteins induced a significant increase of the number of autophagosomes compared to the overexpression of GST, used as a control (Figure 6A/B). Importantly, impairment of IRGM expression leads to a decrease of the number of autophagosomes observed upon overexpression of each of the three viral proteins, as the reduced expression of ATG5 (Figure 6C/D/E/F). Thus, our results show that the MeV-C, HCV-NS3 and HIV-NEF proteins promote autophagosome accumulation via a molecular process involving IRGM.

## Discussion

### Interactome RNA viruses/autophagy

We found that the autophagy network is highly and significantly targeted by RNA viruses suggesting that this cellular process plays an important role in virus biology. Our data suggest that RNA viruses target proteins that although central to autophagy also present a high number of physical connections with proteins involved in other cellular functions. A recent analysis of the global organization of the autophagy network revealed that this conserved cellular function is connected to other cellular functions such as proteolysis, signal transduction, phosphorylation and vesicle transport [35]. Thus, molecular bridges between autophagy and other cellular functions might be preferentially targeted by RNA viruses. The ability to manipulate multifunctional proteins might empower RNA viruses with the ability to fine-tune different complementary cellular functions which are necessary for successful virus infection and replication. A major challenge remains to determine how these viral/cellular protein interactions translate into functional changes imposed by RNA viruses on autophagy and/or other connected cellular functions.

Interaction between viral proteins and autophagy-associated proteins can be explained either by a viral adaptation to this cellular function or alternatively autophagy-associated proteins might be devoted to detection/binding of pathogen proteins to promote anti-viral function. For instance, SQSTM1 (also called p62) brings cargos of ubiquitinated proteins to autophagy degradation [36]. Recently, SQSTM1 was shown to protect mice against Sindbis virus infection by promoting autophagy-dependent but ubiquitination-independent capsid protein degradation [13]. We found here that SQSTM1 has a potential to bind proteins of at least 3 different families of human-infecting RNA viruses. Whether these interactions act in the host cellular defense or are viral subversive pathways has to be tested in specific virus/host cell contexts. GOPC is another highly targeted autophagy-associated protein. However, GOPC appears to be poorly dedicated to autophagy suggesting that its targeting might essentially affect non-autophagy cellular processes.

Only two highly autophagy-dedicated proteins were found to interact with RNA virus proteins, BNIP3 and IRGM. Another targeted autophagy-associated protein, TMEM74 is exclusively connected to the autophagy network, since we found it interacts with BNIP3. BNIP3 and TMEM74 are involved in stress-induced autophagy regulation [37], [38]. Interestingly we found that BNIP3 is able to interact with a large number of proteins within the autophagy network acting at different steps of the autophagic process. This suggests that under specific conditions this protein might allow the coordination and the concerted action of different sub-networks necessary for autophagy progression/inhibition. Whether BNIP3 and TMEM74 are effective during viral infection is unknown, although they might both (co)-regulate cellular responses to viral infection through autophagy induction.

### IRGM, an autophagy-associated protein highly targeted by RNA viruses

We focused our functional work on IRGM since our data support that this protein might be an important regulator of autophagy. We would thus expect that the interaction of a viral protein with IRGM might trigger a functional and specific effect on this cellular function. Many immunity associated GTPases (IRG) exist in mammals and these proteins play an important role in defense against intracellular pathogens [39]. Only two IRG exist in humans, IRGC and IRGM [40]. IRGM is a genetic risk factor in Crohn's disease and tuberculosis [41]–[43]. Human IRGM is constitutively expressed, contrarily to its mouse homolog Irgm1, and it was shown to regulate both IFN-γ- and rapamycin-induced autophagy in human macrophages [27]. Nevertheless the molecular mechanism underlying its function in autophagy remains poorly understood. A recent study reported a role of IRGM in mitochondrial fission important for autophagic control of intracellular mycobacteria [29]. Furthermore loss of tight regulation of IRGM expression compromises the control of intracellular replication of Crohn's disease-associated adherent invasive *Escherichia coli* by autophagy [28]. However, prior to our study no molecular protein connection of IRGM with autophagy has been reported. We found that IRGM is able to directly interact with several autophagy proteins, whereas we did not find any other IRGM interacting human proteins through a yeast two-hybrid screen against a normalized human spleen cDNA library (data not shown). IRGM molecular partners in the autophagy network are involved in the initiation/elongation phases (ATG5, ATG10, MAP1CL3C and SH3GLB1) suggesting that IRGM would modulate the initial steps of autophagy. As previously described [29], we found that IRGM is located in the mitochondrial fraction (Figure S9). This suggests that IRGM interactions with autophagy-associated proteins might take place at the mitochondria, in initial phases of the autophagic process. At least two of the potential IRGM interactors might be partially located at the mitochondria under particular cellular contexts. ATG5 was shown to be able to physical associate with a mitochondria located protein IPS-1/MAVS [44]. Additionally, a fraction of SH3GLB1 is located in the mitochondria and is required for the maintenance of mitochondrial morphology [45].

IRGM is known to play a protective role against bacterial infection favoring IFNγ-mediated autophagy elimination of *Mycobacterium bovis* in macrophages [27] and anti-bacterial autophagy responses in epithelial cells against *Salmonella thyphymurium* [26] and adherent-invasive *Escherichia coli* [28], [46]. However, we found that in the context of RNA virus infections, IRGM does not contribute to a protective role but instead promotes virus replication. Virus/host co-evolution might have lead to subversion of an initial protective mechanism initiated by IRGM/viral protein interaction. Indeed, autophagy induction involving IRGM is ultimately exploited by MeV, HCV and HIV-1 and favours viral infectious particle production. Thus for viruses able to inhibit autophagy maturation, additional molecular virus/autophagy interaction would be necessary to block this specific step. In line with this hypothesis, HIV was recently shown to inhibit autophagosome maturation in 293T and we also found here that HIV-1 may partially inhibit autophagosome maturation in human MDM (Figure S8G). HIV-1-mediated inhibition of autophagy maturation was described to involve HIV-NEF via its interaction with BECN-1 [24]. HCV infection was also reported to prevent autophagosome maturation at an early time of infection ([33] and Figure S8C/D). Alternatively, viral proteins may specifically target IRGM to promote autophagy. Importantly, IRGM-interacting viral proteins MeV-C, HCV-NS3 and HIV-1-NEF, induce autophagy in an IRGM-dependent pathway. Viral proteins might facilitate IRGM interaction with its autophagy partners, by facilitating its relocalization to or stabilization with autophagy-associated proteins involved in the initiation phases of autophagosome formation. Interestingly, the mouse IRGM homolog, Irgm1 was found to bind specific phosphoinositides, through a carboxy (C)-terminal amphipathic helical segment, allowing the recruitment of Irgm1 on nascent and early phagosomes [47].

Although we found that IRGM can interact with influenza A proteins, we found that its absence does not influence influenza A-induced autophagy. Cell infection by influenza A was reported to inhibit autophagy maturation, what we also observed using BAF-treatment (Figure S8E/F) [21]. It is possible that interaction might either not be engaged upon influenza A infection or be involved in other not yet identified IRGM-associated cellular process.

Autophagy is a process that has the potential to degrade pathogens or pathogen-derived molecules trapped within autophagosomes. Viruses and viral proteins are not an exception. Nevertheless its ability to promote cell survival under stress conditions might be beneficial to virus since a major defense mechanism against viral infection is cell death. Overriding this mechanism can give rise to infected cell survival and further viral spread. Furthermore autophagy might provide membranous surfaces required for viral replication. Therefore the molecular analysis of the interplay between viruses and autophagy as well as of its consequences on viral and cellular biology might be of importance to control viral infection. We highlight here and unrevealed role of IRGM in autophagy subversion by RNA viruses. Our semi-global interactome approach opens many doors for a better understanding of the interplay between autophagy and RNA viruses by suggesting many possible molecular targets of RNA viruses among the autophagy-associated proteins.

## Materials and Methods

### Ethics statement

The experiments in this article were performed at Biological Safety Level 2 and 3 in accordance with the regulations set forth by the by the national French committee of genetic (commission de génie génétique). Venous blood from anonymous healthy human volunteers was obtained from the blood bank (Etablissement Français du Sang) in accordance with its guidelines, published in the French Journal Officiel, with informed written consent from each volunteer.

### Plasmids and Gateway cloning

All constructions were performed with a Gateway recombinational cloning system (Invitrogen).

### Autophagy-associated proteins ORFs

Complete cDNA for 35 autophagy-associated proteins were purchased from several providers (Table S2). Most cDNAs were available in a pDONR vector (Gateway technology, Invitrogen). For ATG4D, ATG9A, BECN1, IRGM and ULK1, a PCR product containing *att*B sites was generated. Primers used in Gateway cloning are available upon request. These *att*B-PCR products were used in a BP recombination reaction (Invitrogen).

### Viral ORFs

All 80 viral ORFs used are available in ViralORFfeome ORFeotheque in a gateway pDONR vector [48] (Table S3). For the HIV-1 ORFs NEF, VPR, VIF a PCR product containing *att*B sites was generated.

### Yeast two-hybrid array (autophagy vs autophagy and viruses vs autophagy)

35 autophagy-associated cDNAs were transferred by *in vitro* recombination from a pDONR into both pGBKT7 and pACT2. These constructs were respectively transformed in both bait strain AH109 (Clontech) and prey strain Y187 (Clontech). Viral ORFs (baits) (Table S3) were transferred by *in vitro* recombination from a pDONR into the yeast expression vector pGBKT7 and transformed into the yeast bait strain AH109. Autophagy-associated ORFs (prey) were transferred into pACT2 and transformed into the yeast strain Y187. Yeast cells were mated and subsequently plated on a selective medium lacking histidine to test the interaction-dependent transactivation of the *HIS3* reporter gene.

### Protein-protein interaction from the literature

All binary interactions between human autophagy proteins were extracted from the VirHostNet knowledge base and manually checked in each original paper. Protein-protein interaction network graphics were performed using the networks GUESS tool (http://graphexploration.cond.org).

### Networks metrics

The degree k of a node v in a graph G is the number of edges that are incident to this node. The betweenness b of a node v in a graph G can be defined by the number of shortest paths going through the node v and is normalized by twice the total number of protein pairs in the graph G (n*(n-1)). The equation used to compute betweenness centrality, b(v), for a node v is:where g_ij_ is the number of shortest paths going from node i to j, i and j, V and g_ij_(v) the number of shortest paths from i to j that pass through the node v:
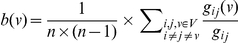



### Interconnectivity significance

The overall statistical significance of the observed autophagy-associated proteins interconnectivity (number of protein-protein interactions) was assessed by a random resampling testing procedure (n = 10,000 permutations).

### Cells

HeLa and GFP-LC3-HeLa cells were maintained in RPMI 1640 supplemented with 0,5 mg/mL gentamicin, 2 mM L-glutamine and 10% fetal calf serum. HEK293T, Huh7.5, GFP-LC3-Huh7.5, A549, GFP-LC3-A549, Vero cells and MDCK were maintained in DMEM supplemented with 0,5 mg/mL gentamicin, 10% fetal calf serum. Additionally Huh7.5 and Huh7.5-GFP-LC3 were supplemented with 1% of non-essential amino acids. Monocytes were purified from the blood of healthy human donors. Human monocytes were cultured in RPMI 1640 supplemented with 10% fetal calf serum and differentiated into macrophages using 10 ng/mL of rh-M-CSF during 6 days (Immunotools, Friesoythe, Germany).

### Transfection

HEK293T cells were transfected using jetPEI (PolyPlus, Illkirch, France) according to manufacturer's instructions. HeLa, GFP-LC3-HeLa, Huh 7.5, GFP-LC3-Huh 7.5 were transfected using lipofectamine 2000 (Invitrogen). A549 and GFP-LC3-A549 were reverse transfected using lipofectamine 2000.

### Antibodies

*Anti-*Glutathione-S-Transferase (GST) peroxidase (A7340), anti-FLAG M2 peroxidase (A8592), anti-MAP1LC3B (L7543), anti-Actin (A2066), anti-Myc (C3956), anti-ATG5 (A0856) and anti-eGFP (G6795) were from Sigma (St Louis, Mo, USA). Anti-IRGM (NT) antibody PK-AB718-4543 was purchased from PromoKine (Heidelberg, Germany) was used for immunofluorescence studies. Anti-IRGM (ab93901) purchased from abcam (Cambridge, UK) was used to detect IRGM by western blot. Anti-ATG5 mouse monoclonal antibody clone 177.19 from Millipore was used to detect endogenous ATG5 by immunofluorescence. Anti-SH3GLB3 mouse monoclonal purchased from Sigma (St Louis, Mo, USA) was use to detect endogenous protein by immunofluorescence. Anti-cytochrome c mouse monoclonal was purchased from BD Biosciences (Le Pont de Claix, France) and the anti-GAPDH mouse monoclonal from Santa Cruz Biotechnology (Santa Cruz, USA). Anti-rabbit HRP (NA 934) was from Amersham Biosciences (Uppsala, Sweden). Anti-mouse Alexa Fluor 568 and 488 was purchased from Invitrogen (Molecular Probes).

### Confocal microscopy

HeLa cells were co-transfected with GFP–IRGM and FLAG tagged ATG10, ATG5, SH3GLB1, MAP1LC3C, MeV-C, HCV-NS3 or HIV-NEF. After 24 h cells were fixed with 2% paraformaldehyde stained using an anti-FLAG antibody followed by secondary antibody conjugated to Alexa Fluor 568. For endogenous IRGM detection by immunofluorescence, MeV infected HeLa cells were fixed in cold acetone and IRGM was detected using an anti-IRGM polyclonal antibody from PromoKine (Heidelberg, Germany) followed by a secondary antibody conjugated to Alexa Fluor 488. Endogenous ATG5 or SH3GLB1 were detected respectively using an anti-ATG5 mouse monoclonal antibody clone 177.19 from Millipore or an anti-SH3GLB3 mouse monoclonal from Sigma (St Louis, Mo, USA). Virus infected GFP-LC3-expressing cells were fixed with 4% paraformaldehyde. Bafilomycin A1 for flux experiments was purchased from Sigma (St Louis, Mo, USA SIGMA). GFP-LC3 HeLa cells and GFP-LC3 A549 were treated for 5 hrs with 100 nM of bafilomycin while GFP-LC3 Huh 7.5 cells were treated for 24 hrs. The number of GFP^+^ vesicles per cell profile was numerated from one single plan section per cell. In each case, number of GFP^+^ vesicules was numerated from 100 to 200 cells for each experiment. All the cells were analyzed using a Axiovert 100 M microscope (Zeiss, Göttingen, Germany) equipped with the LSM 510 system (Zeiss) and observed with a magnification of 63× (oil immersion).

### Co-AP purification

1.5 µg of each expression vector were transfected in HEK293T cells. Cell lysis was done 48 hrs post-transfection. Glutathione-sepharose 4B beads (GE healthcare, Saint Cyr au Mont d'Or, France) were used for the co-AP purification. For the indicated experiments HEK293T cells were transfected with both pCi-neo-3X FLAG and pDESTmyc expression vectors. Protein G sepharose 4B beads coated with 1 µg of anti-Myc antibody were used for a co-AP.

10×10^6^ HeLa cells were transfected or not with pCi-neo-3X FLAG IRGM and infected with MeV (MOI = 1) 24 hrs post-transfection. Cells were lysed 24 hrs post-infection. Protein G sepharose 4B beads coated with 1 µg of anti-FLAG mouse monoclonal antibody were used for a co-AP of the FLAG tagged IRGM. Endogenous ATG5 associated to FLAG-tagged IRGM was detected using anti-ATG5 from Sigma (A0856).

### Small interfering RNA experiments

Smartpool si-ATG5, si-IRGM and control si-RNA were from Dharmacon (Perbio, Brebières, France). 1.10^5^ HeLa, HeLa-GFP-LC3, Huh 7.5, Huh 7.5-GFP LC3 cells were plated in 6-well plates 24 hrs prior to transfection with 100 ρmol si-RNA using Lipofectamine RNAiMAX (Invitrogen) according to manufacturer's instructions. A549 cells and GFP-LC3-A549 were reverse transfected. Human MDM were transfected with 3 µg of si-RNA using Amaxa Human Macrophage Nucleofector Kit according to manufacturer's instructions.

### Preparation of mRNA and cDNA synthesis

Total RNA was extracted from 10×10^6^ cells, isolated by using total RNeasy isolation kit Qiagen and treated with DNAse (DNase Ambion Turbo DNA free, Ambion) to remove genomic DNA according to manufacturers instructions. Oligotex Direct mRNA isolation kit (Qiagen) was used to isolate mRNA from total RNA and cDNA was synthesized using mRNA (0.5 µg) by High Capacity RNA-to-cDNA Master Mix (Applied BioSystems) according to manufacturers instructions.

### mRNA quantification

IRGM mRNA was quantified with a quantitative real-time polymerase chain reaction (qRT-PCR). qRT-PCR reactions were performed with the StepOnePlus Real-Time PCR System (Applied) using the FastStart Universal SYBR Green Master (Rox) (Roche). cDNA was synthesized using the mRNA from the cells as template, and RT^2^ qPCR Primer Assay for Human IRGM (Qiagen) was used. The amount of measured transcripts was normalized to the amount of the Ribosomal protein S9 transcript. Melting curve analysis was performed after each run to analyse specificity of primers. We assessed the presence of contaminating genomic DNA using a minus-reverse transcriptase control in qRT-PCR experiments.

### Endogenous IRGM detection by western blot

10×10^6^ HeLa or Huh 7.5 cells were treated with Smartpool si-IRGM and control si-RNA were from Dharmacon. After mRNA extraction as previously described 4 volumes of cold (−20°C) acetone was added to cell lysates and incubated for 60 min at −20°C. Solution was centrifuged 20 min at 13,000 x *g.* Supernatant was discarded, pellet was dried and ressupended in NuPage LDS sample buffer (Invitrogen) with bond-breaker TCEP solution (Thermo Scientific).

### MeV strain infection and titration

HeLa cells were infected with the MeV Edmonston strain 6 h post-seeding at MOI of 3 for 24 or 48 hrs. Following 5 cycles of freezing at −80°C and defrosting at 37°C total infectious particles were quantified by limiting dilution on confluent Vero cells.

### HCV strain infection and titration

Huh-7.5 cells were infected with HCV JFH1 strain 6 h post-seeding at a MOI of 1. The level of virus particles present in culture supernatants was determined by end-point dilution and Core-specific immunofluorescence staining as described 24 or 48 hrs post-infection [49].

### Influenza A strain, infection and titration

A549 cells were infected with influenza virus A/New Caledonia (H1N1) at a MOI of 0.1. Cell supernatants were harvested at 24 hrs post-infection and samples were titrated by plaque assay (PFU) in MDCK cells under agar overlay.

### HIV-1 strain and infection

HIV-1 infections were performed with normalized amounts of supernatants of R5 HIV-1-transfected cells. MDM were infected with 250 µL of a viral solution containing 170 ng/mL p24 for 2 hrs at 37°C. Cells were then centrifuged during 5 minutes at 1200 rpm and the supernatant was removed. The cells were cultured for 1 day in 1 mL of complete medium containing M-CSF. Infection was followed by measuring HIV-1 gag p24 in the supernatants of the infected cells using a p24 antigen capture ELISA (Innogenetics).

## Supporting Information

Figure S1 **Interconnectivity distribution.** The resampled number of ppi between the 44 proteins of the autophagy network (interconnectivity) was computed n = 10.000 times based on a random resampling procedure. The observed interconnectivity inside the autophagy network (n = 150) appears significantly greater than the maximum number obtained after resampling. Similar interconnectivity was observed excluding our own yeast two-hybrid data set (n = 123).***p<0.0001.
(TIF)

Figure S2 **Autophagy-associated proteins contribute differently to the autophagy network.** Autophagy-associated proteins are plotted according to their context connectivity (A), or context centrality (B), corresponding to the x and y axis of the Figure 2A, respectively.
(TIF)

Figure S3 **Targeting of human autophagy network by different RNA virus families.** (A) Human autophagy network targeted by proteins from the *Paramyxoviridae* family (MeV, Mumps). (B) *Flaviviridae (*Kyasanur forest disease virus, HCV, Tick-borne encephalitis virus. (C) *Orthomyxoviridae* (influenza A). Each node represents one autophagy-associated protein. Black nodes represent autophagy-associated proteins with unknown RNA virus interactions and red nodes represent autophagy-associated proteins targeted by at least oneviral protein from a particular family. Blue edges represent novel ppi. Gray edges represent ppi retrieved from the literature. The width of the nodes is proportional to the degree of the proteins in the autophagy network context, i.e. the number of autophagy-associated proteins directly interacting with the considered protein.
(TIF)

Figure S4 **Interaction of IRGM with RNA viruses proteins.** IRGM was tested by yeast two-hybrid against 83 viral proteins from 5 different RNA virus families. Positive interactions with proteins from viruses of the *Paramyxoviridae, Flaviviridae*, *Orthomyxoviridae*, *Togaviridae* and *Retroviridae* family were found. One experiment representative of three is shown.
(TIF)

Figure S5 **Endogenous ATG5 interacts with overexpressed IRGM.** HeLa cells were transfected with 0.5 µg of expression vector encoding FLAG-SQSTM1 or FLAG-IRGM. Cells were lysed and an affinity purification with an anti-FLAG antibody was performed. A) Affinity purified samples (AP) and total lysate were probed with an anti-ATG5 (A) or with and anti-FLAG antibody (B).
(TIF)

Figure S6 **Endogenous IRGM expression and si-IRGM efficiency.** (A) si-IRGM efficiency on exogenous eGFP IRGM expression in HeLa, Huh7.5 and A549 cells. Cells were transfected with 1.5 µg of expression vector encoding eGFP-IRGM and simultaneously treated with control or IRGM specific si-RNAs for 48 hrs. Cells were lysed and analyzed by western-blot for the expression of eGFP (top panel) and actin (bottom panel) detection. (B) Anti-IRGM antibody recognition of Flag tagged IRGM. HeLa cells were transfected with 0.5 µg of expression vector encoding FLAG-IRGM and simultaneously treated with control or IRGM specific si-RNAs for 24 hrs. Cells were lysed and analyzed by western-blot for the expression of IRGM. (C) Full-length protein gel with size markers showing endogenous IRGM expression in Huh7.5 and A549 cells side to side with overexpressed IRGM silenced by si-IRGM. (D) Huh7.5 cells treated with si-control or si-IRGM. (E) si-IRGM efficiency on endogenous IRGM. HeLa and Huh 7.5 cells were treated with control or IRGM si-RNAs for 24 hrs. Cells were lysed and analyzed by western-blot for IRGM (top panel) and actin (bottom panel) detection. (F) si-IRGM efficiency on endogenous IRGM mRNA. 10×10^6^ cells HeLa, Huh 7.5 and A549 cells were treated with control or IRGM si-RNAs for 24 hrs. Total RNA was extracted, mRNA was isolated and cDNA was synthesized. IRGM mRNA was quantified with a quantitative real-time polymerase chain reaction (qRT-PCR). The amount of measured transcripts was normalized to the amount of the Ribosomal protein S9 transcript. (G) Endogenous IRGM expression (red) was assessed by immunofluorescence in HeLa and Huh 7.5 cells treated with si-control or si-IRGM. Nuclei were labelled with DRAQ5 (blue).
(TIF)

Figure S7 **si-ATG5 efficiency.** (A-B-C) si-ATG5 efficiency in HeLa (A), Huh7.5 (B) and A549 (C) cells. Cells were treated with control or ATG5 si-RNAs for 48 hrs and were either uninfected or infected with MeV (MOI = 3), HCV JFH-1 (MOI = 1) or influenza A/H1N1/New Caledonia (MOI = 1) for 24 hrs. Cells were lysed and analyzed by western-blot for the expression of ATG5 (top panel) and actin (bottom panel) detection.
(TIF)

Figure S8 **MeV, HCV and HIV-1-induced autophagosome accumulation is dependent on IRGM.** (A-B) MeV-induced autophagy is dependent on IRGM. GFP-LC3-HeLa were treated with si-control, si-ATG5 or si-IRGM and either left uninfected or infected with MeV Edmonston (MOI = 3) for 24 hrs. Cells were treated with 100 nM bafilomycin during the last 5 hrs of infection. Autophagy was monitored by evaluating the number of GFP-LC3+ vesicles per cell profile by confocal microscopy. Representative profiles for each condition (A) and the corresponding graph representing the number of GFP-LC3+ vesicles per cell profile (B) are shown, error bars, mean ± SD of duplicates. One experiment representative of three done in duplicate is shown. (C-D) HCV-induced autophagosome accumulation is dependent on IRGM. GFP-LC3-Huh7.5 were treated with si-control, si-ATG5 or si-IRGM and either left uninfected or infected with HCV JFH-1 (MOI = 1) for 24 hrs. Cells were treated with 100 nM bafilomycin during infection. Autophagy was monitored by evaluating the number of GFP-LC3+ vesicles per cell profile by confocal microscopy. Representative profiles for each condition (C) and the corresponding graph representing the number of GFP-LC3+ vesicles per cell profile (D) are shown, error bars, mean ± SD of duplicates. One experiment representative of three done in duplicate is shown. (E-F) Influenza A-induced autophagosome accumulation is not impaired by IRGM absence. GFP-LC3-A549 were treated with si-control, si-ATG5 or si-IRGM and either left uninfected or infected with influenza A/H1N1/New Caledonia (MOI = 1) for 24 hrs. Cells were treated with 100 nM bafilomycin during the last 5 hrs of infection. Autophagy was monitored by evaluating the number of GFP-LC3+ vesicles per cell profile by confocal microscopy. Representative profiles for each condition (E) and the corresponding graph representing the number of GFP-LC3+ vesicles per cell profile (F) are shown, error bars, mean ± SD of duplicates. One experiment representative of three done in duplicate is shown. (G) HIV-1-induced autophagosome accumulation is dependent on IRGM. Monocyte-derived macrophages (MDM) were treated with the indicated si-RNA and were either left uninfected or infected with HIV-1 for 24 hrs. Cells were treated with 100 nM bafilomycin during infection. Cells were lysed and analyzed by western-blot for the detection of LC3-I and LC3-II (top panel) and GADPH (bottom panel). One experiment representative of three is shown with the quantification number representing the intensity of LC3-II/GAPDH bands normalized to the uninfected condition.
(TIF)

Figure S9 **IRGM is located at the mitochondria.** HeLa cell cytosol or mitochondria fractions (Qproteome Mitochondria Isolation Kit) were probed for the presence of IRGM, cytochrome c or GAPDH.
(TIF)

Table S1 **Autophay-associated proteins.** 44 different proteins were considered as autophagy-associated proteins based on the PMID demonstrating protein role in autophagy (third column of the table). The Ensembl gene identifier, official name, synonym(s) and Ensembl description are indicated. Gene names indicated in italic are the usual names found in the literature.
(XLS)

Table S2 **Autophagy-asscociated proteins used in experimental work.** Complete cDNA for 35 autophagy-associated proteins were purchased from several different providers referenced in this table. Ensembl gene identifier, official name, supplier and purchased vector are indicated.
(XLS)

Table S3 **Viral proteins used in experimental work.** GenBank identifier of the viral proteins tested, ViralORFeome clone identifier, viral strain, viral protein domain, virus, viral family are indicated.
(XLS)

Table S4 **Protein/protein interactions of virus/autophagy-associated proteins by yeast two-hybrid array.** Each individual virus/autophagy-associated proteins ppi is represented as an individual line of the table. For each interaction, the autophagy-associated protein official name and Ensembl gene identifier are indicated. GenBank identifier of the viral protein involved in the interaction as well as the ViralORFeome clone identifier, viral strain, viral protein domain, virus, viral family are indicated under the same table line. The PMID of the interactions found in the literature is indicated.
(XLS)

Table S5 **Protein/protein interactions between autophagy-associated proteins by yeast two-hybrid array.** Each individual ppi among autophagy-associated proteins is represented as an individual line of the table. For each interaction, the two autophagy-associated proteins involved are given with their official names and Ensembl gene identifiers. The PMID of the interactions found in the literature is indicated.
(XLS)

Table S6 **Autophagy network metrics.** For each of the autophagy-associated proteins its official name and Ensembl gene identifier, its degree in the human interactome, its adjusted betweenness in the human interactome, its degree in the autophagy network and its adjusted betweenness in the autophagy-network are indicated.
(XLS)
