# Supplementary figures and images for: IRGM Is a Common Target of RNA Viruses that Subvert the Autophagy Network
Source: PLoS Pathog. 2011 Dec 8;7(12):e1002422. doi: 10.1371/journal.ppat.1002422 (PMC3234227; doi:10.1371/journal.ppat.1002422)

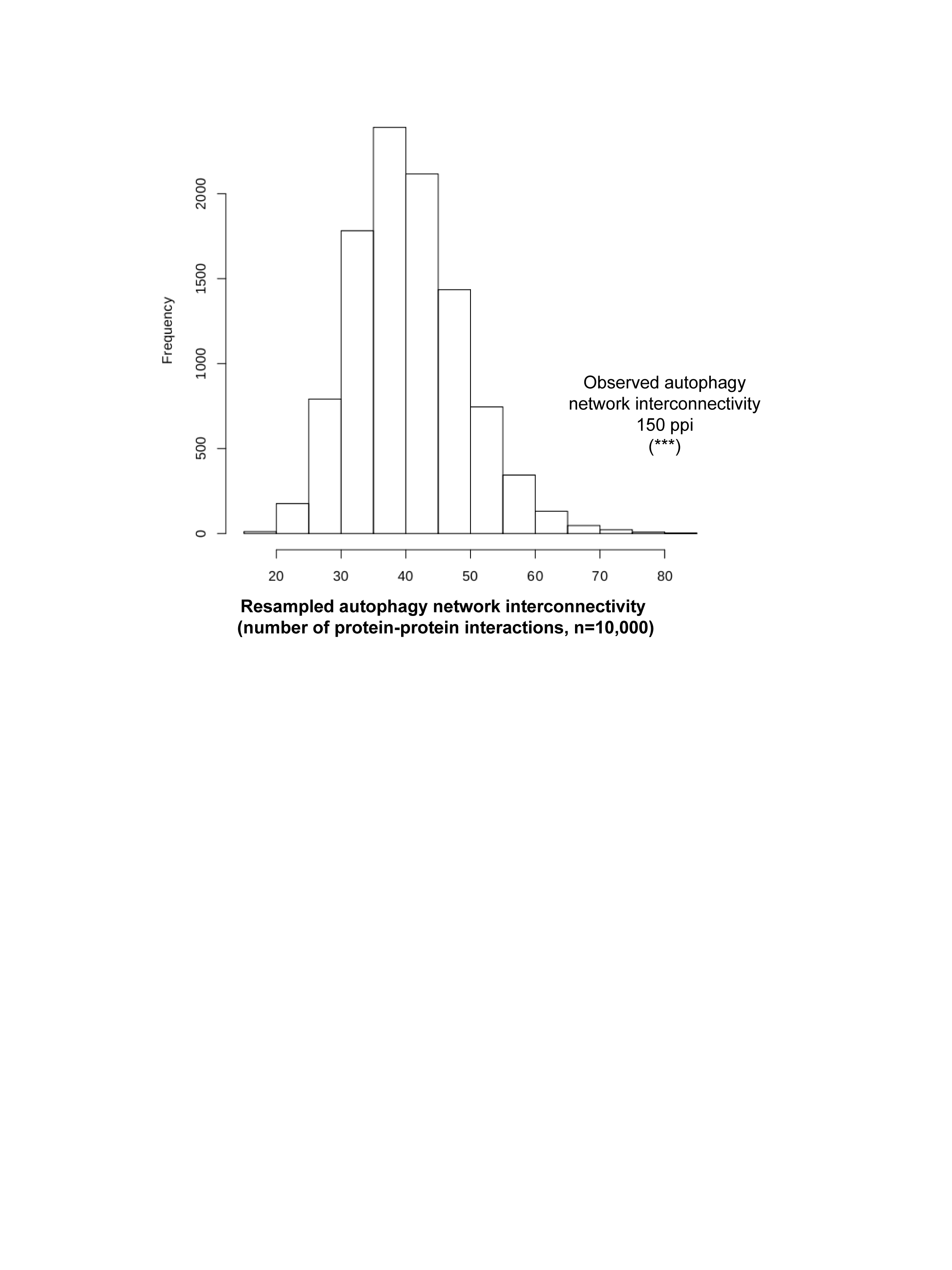

Supplement: Figure S1 — Interconnectivity distribution. The resampled number of ppi between the 44 proteins of the autophagy network (interconnectivity) was computed n = 10.000 times based on a random resampling procedure. The observed interconnectivity inside the autophagy network (n = 150) appears significantly greater than the maximum number obtained after resampling. Similar interconnectivity was observed excluding our own yeast two-hybrid data set (n = 123).***p<0.0001. (TIF) [file ppat.1002422.s001.tif]

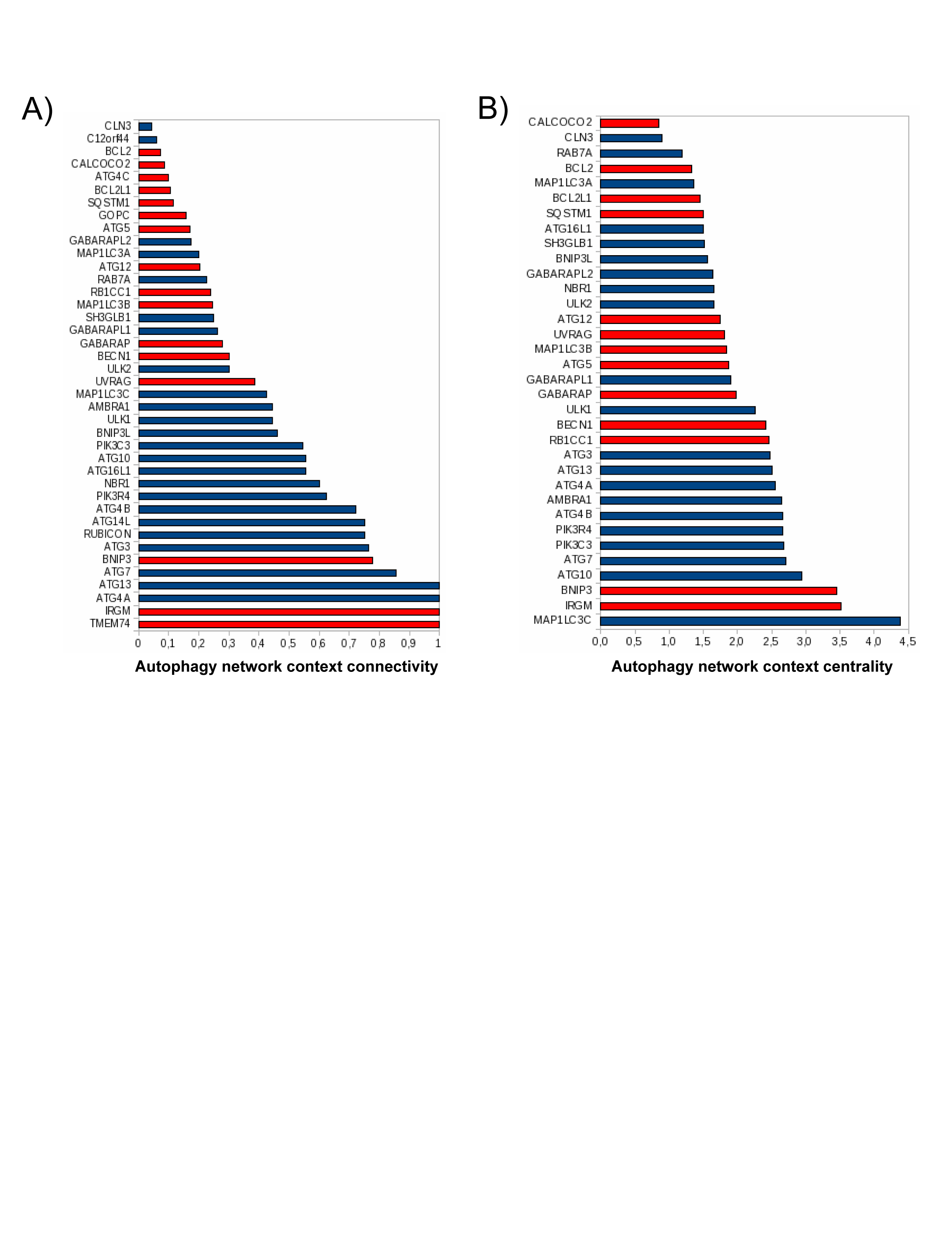

Supplement: Figure S2 — Autophagy-associated proteins contribute differently to the autophagy network. Autophagy-associated proteins are plotted according to their context connectivity (A), or context centrality (B), corresponding to the x and y axis of the Figure 2A, respectively. (TIF) [file ppat.1002422.s002.tif]

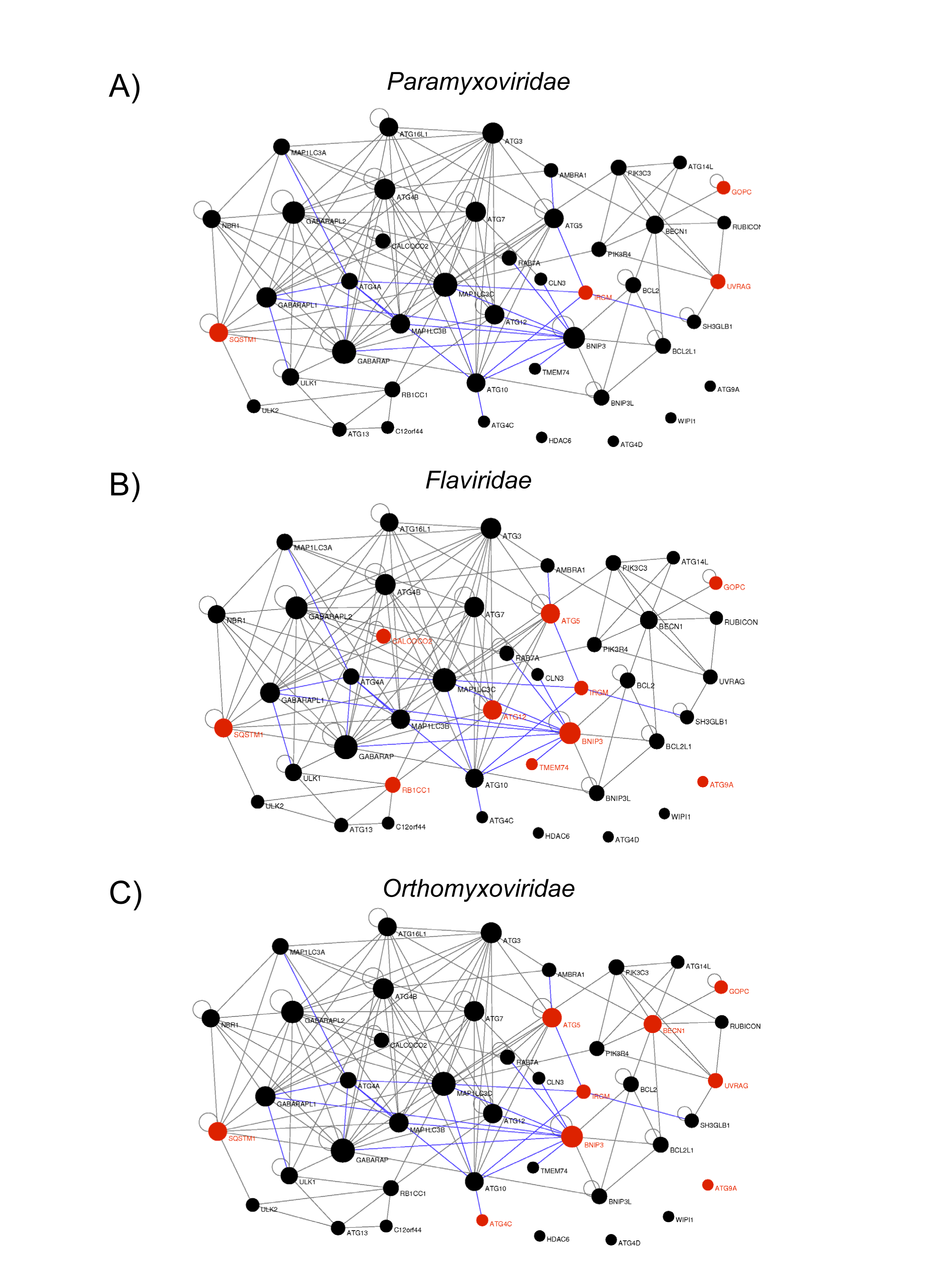

Supplement: Figure S3 — Targeting of human autophagy network by different RNA virus families. (A) Human autophagy network targeted by proteins from the Paramyxoviridae family (MeV, Mumps). (B) Flaviviridae (Kyasanur forest disease virus, HCV, Tick-borne encephalitis virus. (C) Orthomyxoviridae (influenza A). Each node represents one autophagy-associated protein. Black nodes represent autophagy-associated proteins with unknown RNA virus interactions and red nodes represent autophagy-associated proteins targeted by at least oneviral protein from a particular family. Blue edges represent novel ppi. Gray edges represent ppi retrieved from the literature. The width of the nodes is proportional to the degree of the proteins in the autophagy network context, i.e. the number of autophagy-associated proteins directly interacting with the considered protein. (TIF) [file ppat.1002422.s003.tif]

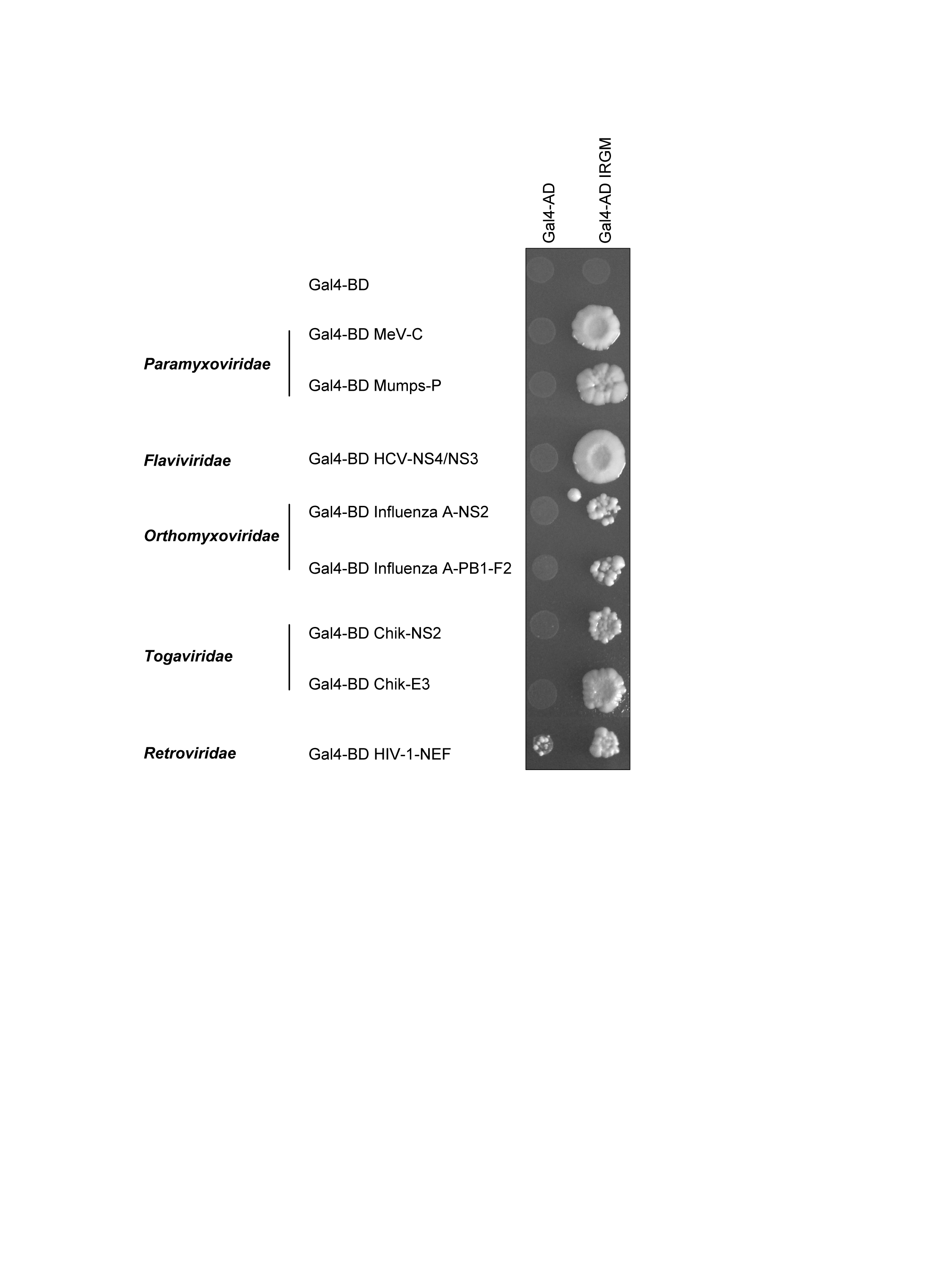

Supplement: Figure S4 — Interaction of IRGM with RNA viruses proteins. IRGM was tested by yeast two-hybrid against 83 viral proteins from 5 different RNA virus families. Positive interactions with proteins from viruses of the Paramyxoviridae, Flaviviridae, Orthomyxoviridae, Togaviridae and Retroviridae family were found. One experiment representative of three is shown. (TIF) [file ppat.1002422.s004.tif]

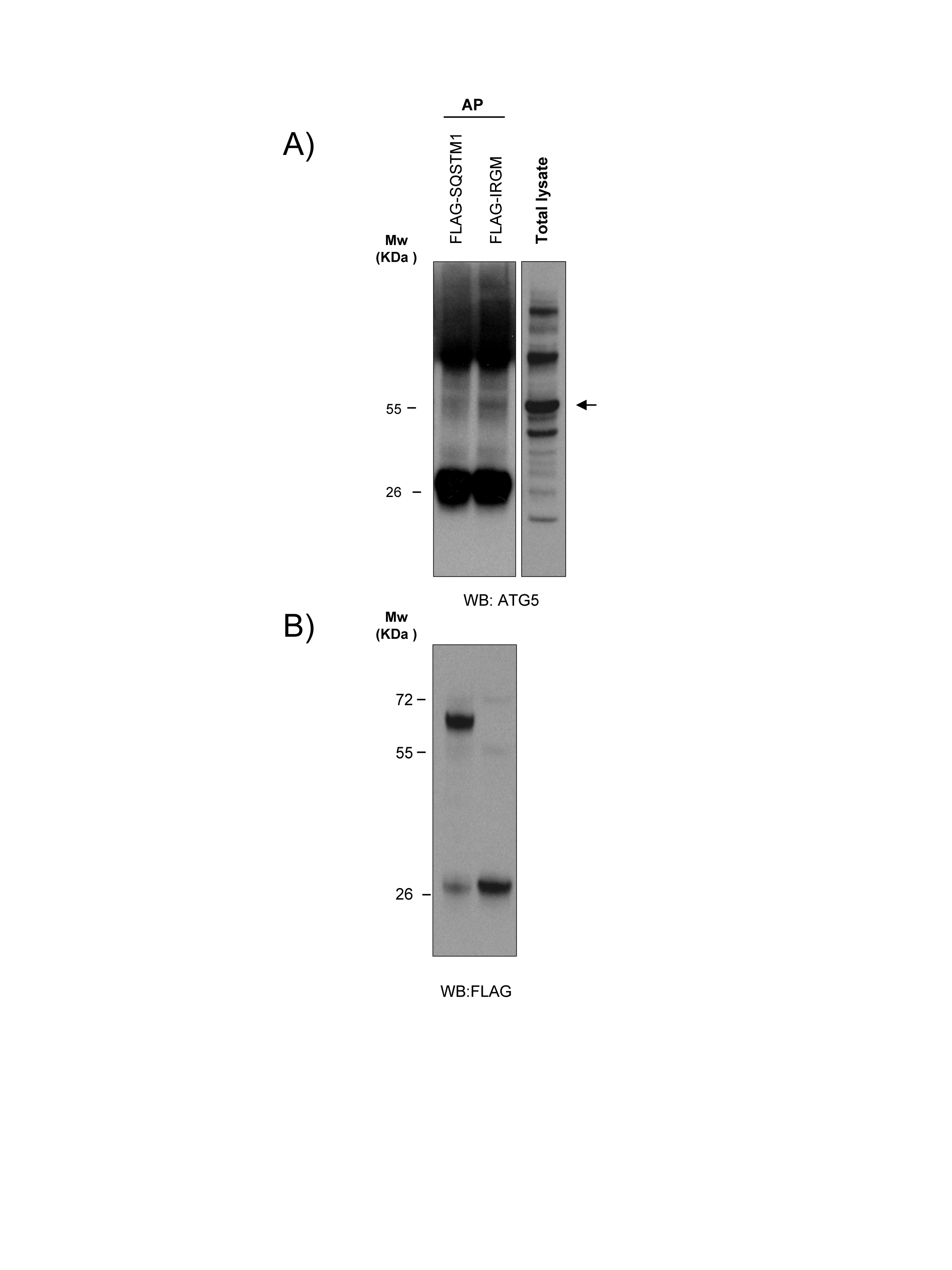

Supplement: Figure S5 — Endogenous ATG5 interacts with overexpressed IRGM. HeLa cells were transfected with 0.5 µg of expression vector encoding FLAG-SQSTM1 or FLAG-IRGM. Cells were lysed and an affinity purification with an anti-FLAG antibody was performed. A) Affinity purified samples (AP) and total lysate were probed with an anti-ATG5 (A) or with and anti-FLAG antibody (B). (TIF) [file ppat.1002422.s005.tif]

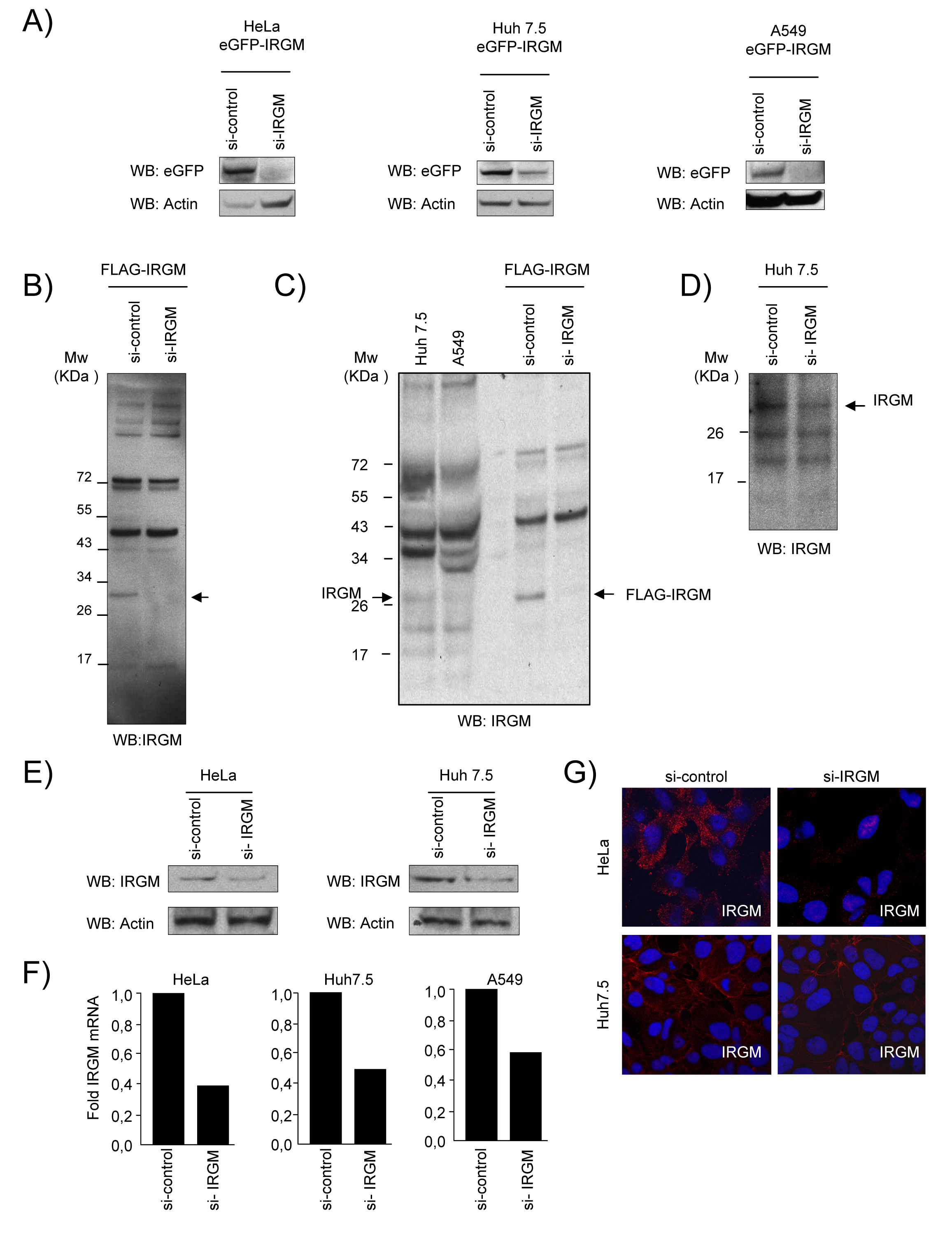

Supplement: Figure S6 — Endogenous IRGM expression and si-IRGM efficiency. (A) si-IRGM efficiency on exogenous eGFP IRGM expression in HeLa, Huh7.5 and A549 cells. Cells were transfected with 1.5 µg of expression vector encoding eGFP-IRGM and simultaneously treated with control or IRGM specific si-RNAs for 48 hrs. Cells were lysed and analyzed by western-blot for the expression of eGFP (top panel) and actin (bottom panel) detection. (B) Anti-IRGM antibody recognition of Flag tagged IRGM. HeLa cells were transfected with 0.5 µg of expression vector encoding FLAG-IRGM and simultaneously treated with control or IRGM specific si-RNAs for 24 hrs. Cells were lysed and analyzed by western-blot for the expression of IRGM. (C) Full-length protein gel with size markers showing endogenous IRGM expression in Huh7.5 and A549 cells side to side with overexpressed IRGM silenced by si-IRGM. (D) Huh7.5 cells treated with si-control or si-IRGM. (E) si-IRGM efficiency on endogenous IRGM. HeLa and Huh 7.5 cells were treated with control or IRGM si-RNAs for 24 hrs. Cells were lysed and analyzed by western-blot for IRGM (top panel) and actin (bottom panel) detection. (F) si-IRGM efficiency on endogenous IRGM mRNA. 10×106 cells HeLa, Huh 7.5 and A549 cells were treated with control or IRGM si-RNAs for 24 hrs. Total RNA was extracted, mRNA was isolated and cDNA was synthesized. IRGM mRNA was quantified with a quantitative real-time polymerase chain reaction (qRT-PCR). The amount of measured transcripts was normalized to the amount of the Ribosomal protein S9 transcript. (G) Endogenous IRGM expression (red) was assessed by immunofluorescence in HeLa and Huh 7.5 cells treated with si-control or si-IRGM. Nuclei were labelled with DRAQ5 (blue). (TIF) [file ppat.1002422.s006.tif]

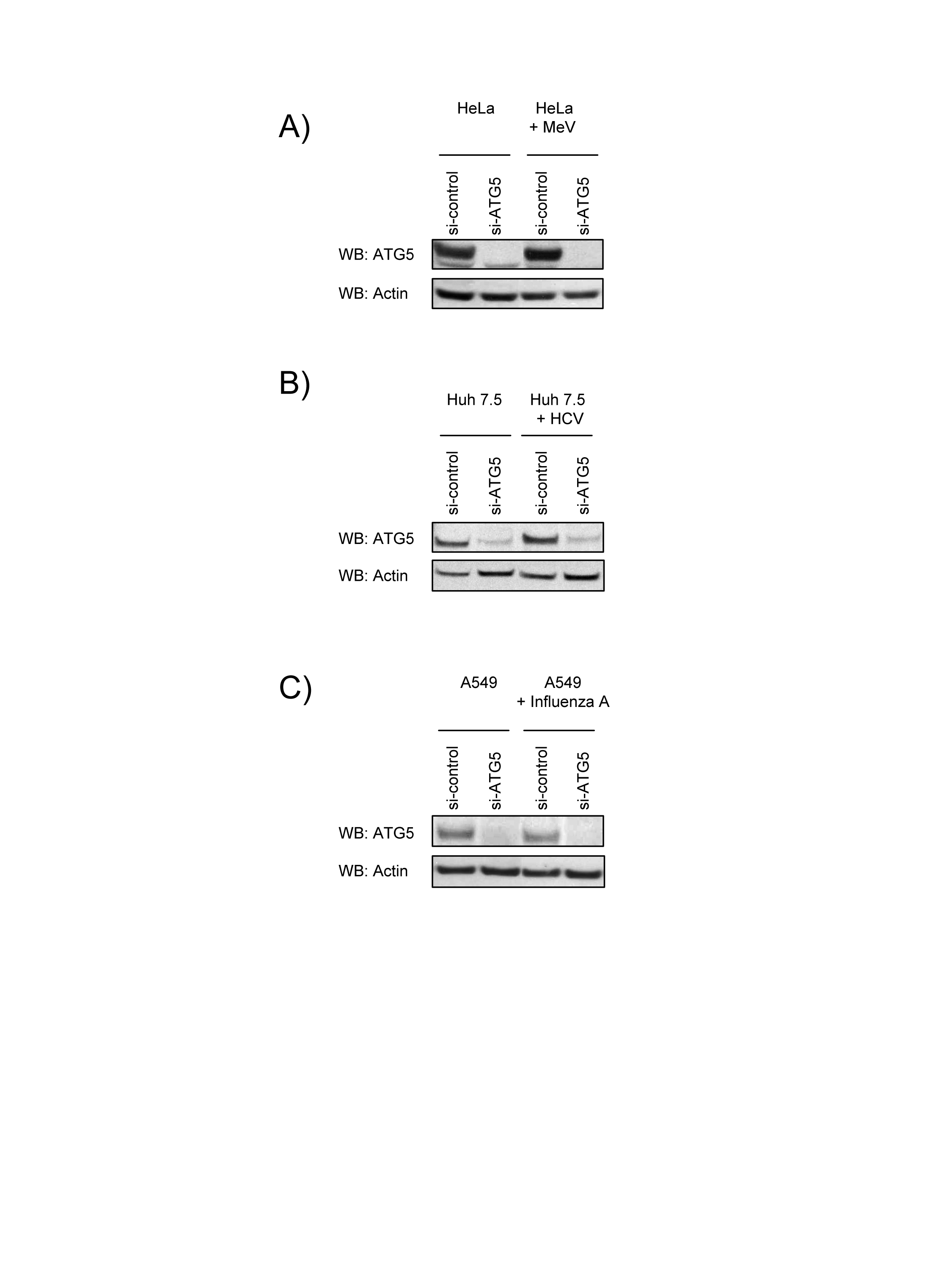

Supplement: Figure S7 — si-ATG5 efficiency. (A-B-C) si-ATG5 efficiency in HeLa (A), Huh7.5 (B) and A549 (C) cells. Cells were treated with control or ATG5 si-RNAs for 48 hrs and were either uninfected or infected with MeV (MOI = 3), HCV JFH-1 (MOI = 1) or influenza A/H1N1/New Caledonia (MOI = 1) for 24 hrs. Cells were lysed and analyzed by western-blot for the expression of ATG5 (top panel) and actin (bottom panel) detection. (TIF) [file ppat.1002422.s007.tif]

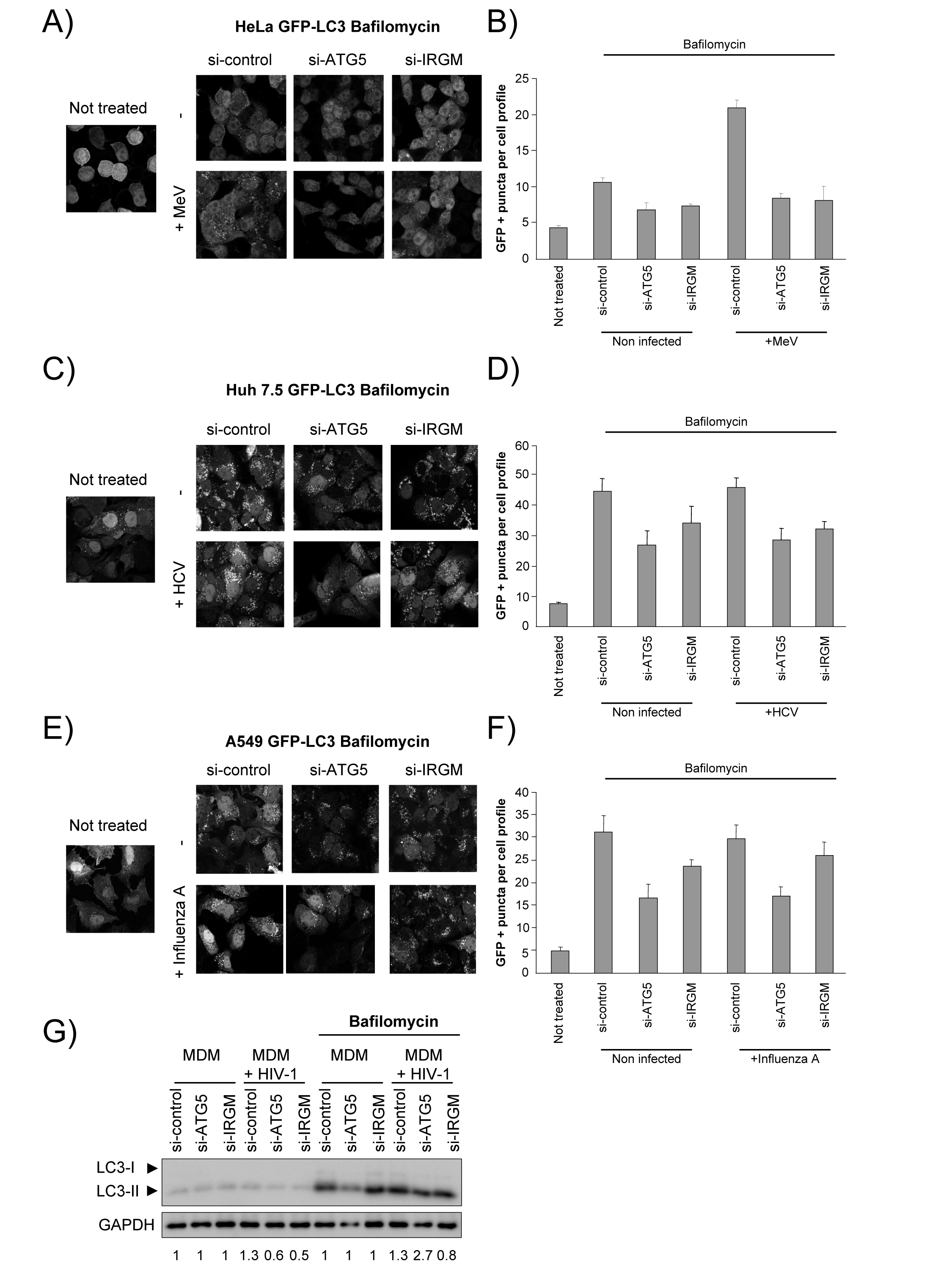

Supplement: Figure S8 — MeV, HCV and HIV-1-induced autophagosome accumulation is dependent on IRGM. (A-B) MeV-induced autophagy is dependent on IRGM. GFP-LC3-HeLa were treated with si-control, si-ATG5 or si-IRGM and either left uninfected or infected with MeV Edmonston (MOI = 3) for 24 hrs. Cells were treated with 100 nM bafilomycin during the last 5 hrs of infection. Autophagy was monitored by evaluating the number of GFP-LC3+ vesicles per cell profile by confocal microscopy. Representative profiles for each condition (A) and the corresponding graph representing the number of GFP-LC3+ vesicles per cell profile (B) are shown, error bars, mean ± SD of duplicates. One experiment representative of three done in duplicate is shown. (C-D) HCV-induced autophagosome accumulation is dependent on IRGM. GFP-LC3-Huh7.5 were treated with si-control, si-ATG5 or si-IRGM and either left uninfected or infected with HCV JFH-1 (MOI = 1) for 24 hrs. Cells were treated with 100 nM bafilomycin during infection. Autophagy was monitored by evaluating the number of GFP-LC3+ vesicles per cell profile by confocal microscopy. Representative profiles for each condition (C) and the corresponding graph representing the number of GFP-LC3+ vesicles per cell profile (D) are shown, error bars, mean ± SD of duplicates. One experiment representative of three done in duplicate is shown. (E-F) Influenza A-induced autophagosome accumulation is not impaired by IRGM absence. GFP-LC3-A549 were treated with si-control, si-ATG5 or si-IRGM and either left uninfected or infected with influenza A/H1N1/New Caledonia (MOI = 1) for 24 hrs. Cells were treated with 100 nM bafilomycin during the last 5 hrs of infection. Autophagy was monitored by evaluating the number of GFP-LC3+ vesicles per cell profile by confocal microscopy. Representative profiles for each condition (E) and the corresponding graph representing the number of GFP-LC3+ vesicles per cell profile (F) are shown, error bars, mean ± SD of duplicates. One experiment representati [file ppat.1002422.s008.tif]

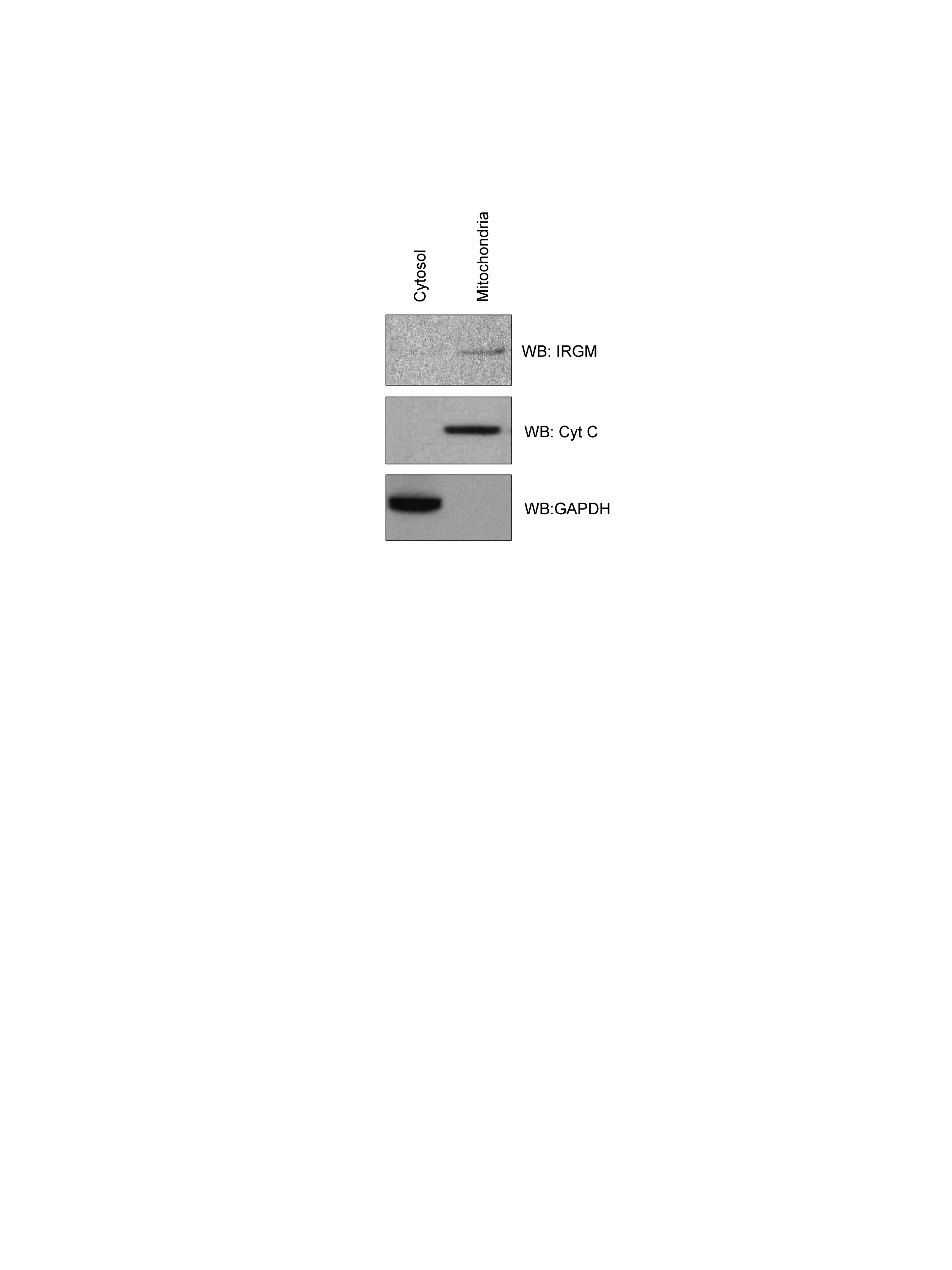

Supplement: Figure S9 — IRGM is located at the mitochondria. HeLa cell cytosol or mitochondria fractions (Qproteome Mitochondria Isolation Kit) were probed for the presence of IRGM, cytochrome c or GAPDH. (TIF) [file ppat.1002422.s009.tif]
